# FER kinase governs invasive growth of head and neck squamous cell carcinoma through dynamic control of growth factor receptor activity

**DOI:** 10.1016/j.neo.2025.101241

**Published:** 2025-10-19

**Authors:** Peter D. Haughton, Lotte N.F.L. Enserink, Sandra Tavares, Wisse Haakma, Garik Galustjan, Sjors Koppes, Lorenza Casasanta, Else Driehuis, Hans Clevers, Yanchun Zhang, Gaofeng Fan, Stefan Willems, Xiaobao Yang, Patrick W.B. Derksen

**Affiliations:** a Department of Pathology, University Medical Center Utrecht, Utrecht, the Netherlands; b Hubrecht Institute, Royal Netherlands Academy of Arts and Sciences (KNAW) and UMC Utrecht, 3584 CT Utrecht, the Netherlands; c Institute of Human Biology (IHB), Roche Pharmaceutical Research and Early Development, Basel, Switzerland; d School of Life Science and Technology, ShanghaiTech University, Shanghai, China; e Department Pathology and Medical Biology, University Medical Center Groningen, Groningen, the Netherlands; f Gluetacs Therapeutics (Shanghai) Co., Ltd

**Keywords:** FER kinase, FER PROTAC, Growth factor receptors, EFGR, MET, Head and neck cancer, Invasion, Inhibition, Patient-derived organoids

## Abstract

Promiscuous activation of growth factor receptors drives sustained MAP kinase signaling, which reinforces oncogene addiction in HPV-negative head and neck squamous cell carcinoma (HNSCC). This feature promotes invasive growth, complicating surgical resection and contributing to high rates of local relapse and poor patient outcomes. Current treatment strategies for locally advanced or non-resectable tumors targeting single growth factor receptors offer limited therapeutic benefit, underscoring the need for alternative targets. Using patient-derived tumor organoid (PDO) models of invasive HNSCC, we demonstrate that FER, a non-receptor tyrosine kinase that correlates with poor survival in HNSCC patients, is essential for growth factor receptor dependent invasive growth in Collagen-I extracellular matrix (ECM) networks. In this setting, FER promotes phosphorylation of EGFR-Y1068 and MET-Y1234/5. Additionally, FER controls ligand-dependent endocytic transport velocity, demonstrating a multifactorial regulation of proximal GFR activation during HNSCC invasion. Finally, genetic loss of function experiments or a FER-specific PROteolysis-TArgeting Chimera (PROTAC) strategy in PDO-based xenograft mouse models, demonstrate that FER is essential for invasive growth and metastasis of HNSCC. In sum, we propose that FER is an indiscriminate regulator of proximal GFR activation in HNSCC, a mechanism that may foster oncogene addition, thereby leading to invasive growth and metastasis. Based on its oncogenic roles and correlations with poor patient prognosis, we nominate FER as a potential candidate for targeted clinical intervention of HNSCC.

## Highlights

• FER, a non-receptor tyrosine kinase, is prognostic in HPV-negative HNSCC and essential for growth factor receptor–dependent invasion.
• In patient-derived tumor organoid models, FER controls MET and EGFR oncogenic signaling required for invasion in 3D Collagen-I extracellular matrix.
• FER regulates ligand-dependent endocytic transport dynamics during HNSCC progression.
• Genetic FER loss or FER-targeted PROTAC therapy suppresses invasion and metastasis in PDO-based xenograft models.
• FER represents a promising therapeutic target for clinical intervention in aggressive HNSCC.

**Significance**

The findings in the current manuscript establish FER, a critical driver of invasive cancer growth, as a promising target for therapeutic intervention with the potential to improve clinical outcomes in HNSCC.

Alt-text: Unlabelled box

## Introduction

Head and neck squamous cell carcinoma (HNSCC) accounts for 90 % of all head and neck cancers [1]. HNSCC is strongly associated with HPV-negative risk factors such as alcohol and tobacco consumption, and is characterized by extensive local invasion into the surrounding tissues [2,3]. Approximately 50 % of all HNSCC patients succumb to the disease within 5 years due to these loco-regional relapses and local/distant metastasis [4]. Although recent improvements in multimodal therapy have marginally improved overall survival, surgery remains a main and primary option, often resulting in cosmetic disfigurement and impaired functionality. To this end, the lack of successful clinical interventions highlights the unmet need to identify the mechanisms that drive the progression of this mutilating disease.

Studies in several cancer types have identified feline sarcoma-related protein (FER), a ubiquitously expressed non-receptor tyrosine kinase, as a mediator of proliferation, cell-cell adhesion, cytoskeleton dynamics and migration [[5], [6], [7], [8], [9], [10]]. FER consists of a phospholipid-binding and membrane targeting amino N-terminal FER/CIP4/Bin/Amphiphysin/Rvs and coiled-coil region, collectively termed an F-BAR domain, followed by an SH2 and a tyrosine kinase domain [11]. Preclinical data implicate FER as a regulator of microtubule dynamics and Dynactin-2–dependent cargo transport, as well as a strong predictor of clinical responses to taxane-containing chemotherapies in triple-negative breast cancer (TNBC) [5,12]. Notably, FER is key to the rapid endocytic recycling of integrin α6 and β1 in high-grade breast cancer during invasive growth and can dictate endosomal sorting of the epidermal growth factor receptor (EGFR) through tyrosine phosphorylation of PKCδ-Y374 [5,13,14]. Importantly, the endocytic pathway provides an additional level of GFR activity, controlling tumor proliferation, invasion and metastasis [15,16]. Both EGFR and MET, GFRs that share similar signaling pathways, localize to endosomal compartments where they can induce MAPK signaling [[17], [18], [19], [20]]. In parallel, approximately 25 % of these receptors are redistributed to the cell surface, augmenting their activity [21,22]. However, it remained unclear if there is a unifying factor or mechanism that controls the promiscuous activation and endosomal transport of the GFRs that drive oncogenic addiction, and if so, if such a mechanism provides options for targeted inhibition.

In HNSCC, EGFR is overexpressed in 90 % of patients [23]. Although cetuximab, a monoclonal antibody targeting EGFR, is approved for clinical management, it demonstrates only a modest response rate of 13 % [24]. EGFR drives invasive growth in HNSCC via ligand-induced dimerization and subsequent autophosphorylation, which triggers effector PI3K-AKT and MAP kinase pathways [25,26]. Primary and acquired resistance to EGFR inhibition have been directly linked to overexpression of MET, and it is well established that EGFR and MET share similar pathway trajectories and phenotypical outcomes in cancer [[27], [28], [29], [30]]. Similar to EGFR, MET and its cognate ligand hepatocyte growth factor (HGF), are overexpressed in approximately 80 % of HNSCC and have been linked to poor survival and disease progression [31]. As a result of the functional overlap between both GFR signaling pathways and the underlying oncogenic addiction, the efficacy of targeting GFRs with single agents seems inherently limited.

In this study we have identified the non-receptor tyrosine kinase FER as a regulator of oncogenic GFR activation in HNSCC. A combination of patient-derived organotypic and xenograft HNSCC models demonstrates that FER may underpin promiscuous activation of GFRs and thus propel resistance towards targeted GFR intervention. Lastly, our preclinical results in PDO and mouse models using targeted proteasomal degradation of FER, advocate FER as a clinical target for tailored inhibition in HNSCC.

## Results

### High FER expression is prognostic and controls invasive growth in HNSCC

**Fig. 1:**
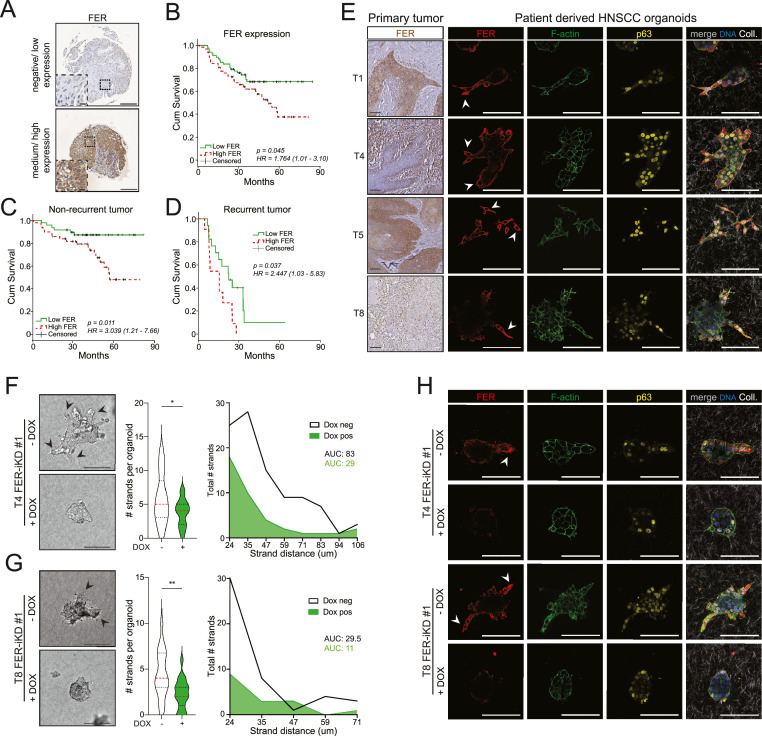
FER kinase drives the invasive growth of HNSCC organoid models and is prognostic in HNSCC A) Representative immunohistochemistry examples of negative/low (top) and medium/high expression (bottom) of FER kinase expression from a HNSCC tissue microarray (TMA). Scale bars: 200 µm. Insets: 20 µm. B - D) High FER expression associates with poor outcomes in HNSCC. Kaplan-Meier survival curves according to FER expression status (HR: 1.764, CI = 1.01 – 3.10, p-value 0.045) (B), non-recurrent tumors (HR: 3.039, CI = 1.21 – 7.66, p-value 0.011) (C), or recurrent tumor burden (HR: 2.447, CI = 1.03 – 5.83, p-value 0.037) (D). E) FER is expressed in invasive HNSCC. HNSCC primary tissue sections and their corresponding PDOs were analyzed for FER expression using immunohistochemistry (left panels, primary tumor). The corresponding HNSCC PDO models were analyzed for FER (red), F-actin (green), and p63 (yellow) expression using immunofluorescence. DNA (blue) and Collagen-I signals (white) are added in the merged image (right panels). White arrowheads indicate invasive strands/cells expressing FER. Scale bars: 100 µm. F - H) FER is essential for invasion. Brightfield images show the effect of FER knockdown (+DOX) in T4 (F) and T8 (G). Black arrowheads indicate invasive strands. Scale bars: 50 µm. The number of invasive strands per organoid (*n* = 30 PDOs per condition, middle violin plot) and the distance of invasive strands (*n* = 20 PDOs per condition, right line graph) were quantified from three independent experiments. Error bars indicate SD; *p < 0.05, **p < 0.01. The Area under the curve (AUC) was employed to quantify differences in strand length between conditions. H) Immunofluorescence images show the effect of FER depletion on FER expression (red), F-actin (green), and p63 (yellow). Merged images (right panels) also depict Collagen-I (white) and DNA (blue). White arrowheads in the left panels indicate FER expression in invasive strands. Scale bars: 100 µm.

To establish a clinical relevance of FER in HNSCC, we investigated the association between FER protein expression levels and patient survival in a tissue microarray (TMA) consisting of 129 HNSCC samples (Table 1). Immunohistochemistry (IHC) was performed with a validated monoclonal antibody and FER expression was scored based on two categories: negative/low, or medium/high expression (Fig. 1A). Analysis of the clinicopathological parameters such as primary tumor size, TNM (stages III, IVA, and IVB), perineural growth or tumor recurrence showed no correlation with FER kinase expression (Table 2). However, high FER expression was significantly associated with lymph node involvement (p-value = 0.01) and lower survival in HNSCC patients (HR: 1.764, CI = 1.01 – 3.10, p-value = 0.045) (Table 2 and Fig. 1B, respectively). Furthermore, high FER expression correlated with decreased patient survival in both non-recurrent (HR: 3.039, CI = 1.21 – 7.66, p-value = 0.011) and recurrent (HR: 2.447, CI = 1.03 – 5.83, p-value = 0.037) HNSCC patients (Fig. 1C and D). In short, high expression of FER is a common feature and a candidate prognostic marker for poor prognosis in HNSCC.

**Table 1:** HNSCC TMA clinicopathological characteristics.

Feature	Patients	
	NO.	%
Age (years)		
≤60	78	60.5
>60	51	39.5
Sex		
Male	86	66.7
Female	41	31.8
UKN	2	1.6
T-primary tumor size		
T1	3	2.3
T2	30	23.3
T3	38	29.5
T4a	40	31.0
T4b	18	14.0
N- lymph node involvement		
N0	23	17.8
N1	21	16.3
N2a	10	7.8
N2b	44	34.1
N2c	24	18.6
N3	6	4.7
Nx	1	0.8
TNM stage		
III	28	21.7
IVA	80	62.0
IVB	21	16.3
Tumor location		
Oropharynx	59	45.7
Hypopharynx	45	34.9
Larynx	25	19.4
Perineural growth		
No	106	84.8
Yes	12	9.6
NA	7	5.6
Recurrent tumor		
No	98	76.0
Yes	28	21.7
NA	3	2.3

**Table 2:** FER kinase association with clinicopathological features.

Feature	Patients			
	FER Negative/low N (%)	FER High/medium N (%)	Pearson Correlation	p-value
T-primary tumor size				
T1	1 (33.3)	2 (66.7)	0.071	0.426
T2	17 (56.7)	13 (43.3)		
T3	21 (55.3)	17 (44.7)		
T4a	21 (52.5)	19 (47.5)		
T4b	7 (38.9)	11 (61.1)		
N- lymph node involvement				
N0	7 (30.4)	16 (69.6)	−0.227	0.01
N1	9 (42.9)	12 (57.1)		
N2a	7 (70)	3 (30)		
N2b	26 (59.1)	18 (40.9)		
N2c	14 (58.3)	10 (41.7)		
N3	4 (66.7)	2 (33.3)		
Nx	0 (0)	1 (100)		
TNM stage				
III	12 (42.9)	16 (57.1)	−0.016	0.857
IVA	46 (57.5)	34 (42.5)		
IVB	9 (42.9)	12 (57.1)		
Tumor location				
Oropharynx	29 (49.2)	30 (40.8)	−0.095	0.285
Hypopharynx	22 (48.9)	23 (51.1)		
Larynx	16 (64)	9 (36)		
Perineural growth				
No	55 (51.9)	51 (48.1)	−0.031	0.728
Yes	4 (33.3)	8 (66.7)		
NA	4 (57.1)	3 (42.9)		
recurrent tumor				
No	49 (50)	49 (50)	0.055	0.536
Yes	17 (60.7)	11 (39.3)		
NA	1 (33.3)	2 (66.7)		

Next, we assessed FER protein expression in our recently established patient-derived HNSCC organoid (PDO) models which show invasion in 3D Collagen-I matrices [32,33] and their corresponding primary tissue sections. A combination of IHC and immunofluorescence (IF) showed that FER is expressed in all invasive cells independent of invasion modes (collective or single cell), suggesting that it may regulate invasion in HNSCC (Fig. 1E). To investigate the functional impact of FER-dependent invasive growth in HNSCC, we generated stable FER knockdown (shRNA) based on established sequences [5,12] using a doxycycline (DOX)-inducible systems in T4 and T8, PDO models with high FER expression (Supplemental Fig. 1A and 1B). FER loss resulted in overt inhibition of invasion in both models (Fig. 1F and G, and Supplemental Fig. 1C and 1D). IF analysis confirmed that FER expression is predominantly localized to the invasive strands in both T4 and T8, and that depletion of FER does not impact basal differentiation based on the marker p63 (Fig. 1H). We also noted an approximate 25 % reduction in cell proliferation upon FER knockdown (Figures 1E and 1F), an expected and known impact of FER inhibition [5,8,12]. To confirm the pro-invasive role of FER and exclude off-target effects of the inducible knockdown system, we reconstituted FER-depleted T4 cells with a V5-tagged, non-targetable, full-length FER cDNA using a lentiviral DOX-inducible system. Indeed, reconstitution using this tool restores proliferation and the invasive phenotype upon FER knockdown, indicating that the observed effects are specific to FER (Supplemental Fig. 1G–1I).

### FER and growth factor receptor expression at the invasive front

**Fig. 2:**
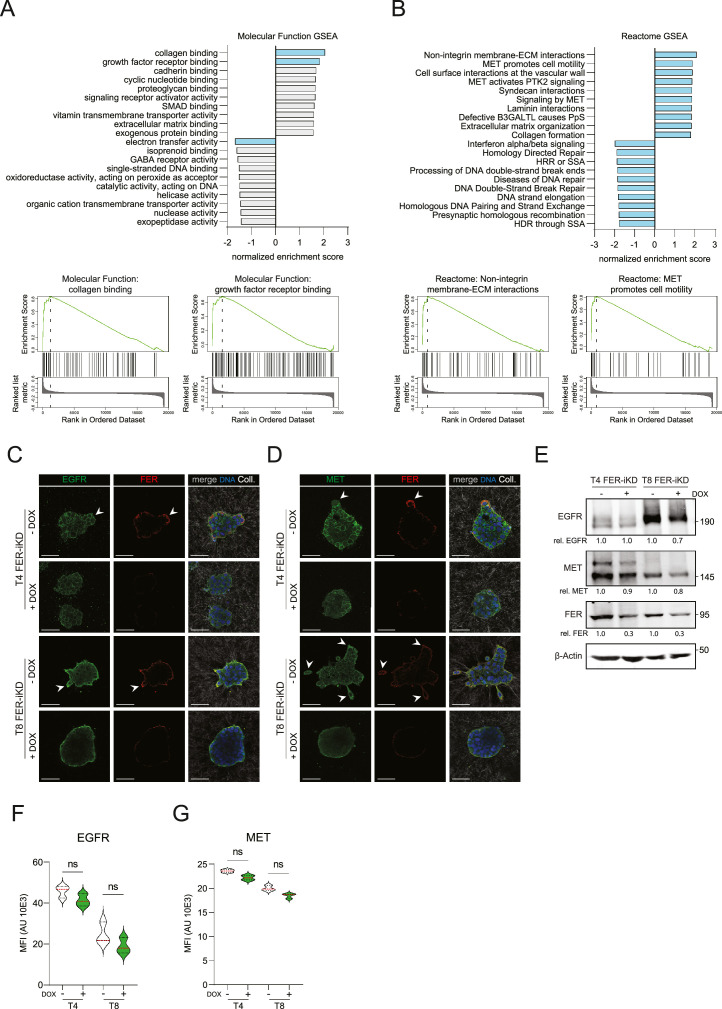
FER regulates GFR activity in HNSCC A and B) Gene Set Enrichment Analysis (GSEA) based on single-cell mRNA sequencing from the T4 FER-iKD PDO model seeded in Collagen-I identifies molecular pathways associated with FER expression. GO Molecular Function pathways (upper panel A) and Reactome pathways (upper panel B) are displayed in bar charts, with significant pathways (false discovery rate, FDR < 0.05) highlighted in blue. X-axis indicates the normalized enrichment score (NES) and y-axis the pathways. Lower panels in (A) and (B) show the top two enriched pathways. C and D) The impact of FER knockdown in T4 or T8 cells (+DOX) on EGFR (green, C) or MET receptor expression (green, D), as well as FER expression (red, middle panels) is shown. Merged images depict the location of the Collagen-I matrix (white) and nuclear DNA (right panels). Arrowheads (white) indicate colocalization of FER with EGFR or MET expression. Scale bars: 100 µm. E) Western blot analysis of FER-iKD HNSCC PDO models probed for EGFR, MET and FER. β-Actin was used as the loading control. Relative FER (FER/β-Actin) and EGFR or MET (GFR/β-Actin) expression are quantified below the panels. F and G) GFR membrane expression analysis upon FER knockdown using FACS analysis. Shown are cell surface expression levels of EGFR (F) and MET (G). Violin plots depict mean fluorescence intensity (MFI) from three independent experiments. Error bars indicate SD; ns indicates non-significant.

Next, we investigated the transcriptional impact of FER depletion in HNSCC PDOs cultured under pro-invasive conditions (Collagen-I). Differential expression (DE) analysis of T4 FER iKD cells followed by Gene Set Enrichment Analysis (GSEA) identified two Gene Ontology (GO) Molecular Function pathways significantly enriched with FER expression: ‘collagen binding’ (NES = 2.06, false discovery rate (FDR) = 0) and ‘growth factor receptor binding’ (NES = 1.84, FDR < 0.006) (Fig. 2A and Supplemental Table 1 and 2). Interestingly, FER knockdown results in reduced mRNA levels of VEGF ligands (*VEGFA* and *VEGFC*) and several EGFR ligands (*AREG, EREG, HBEGF* and *TGFA*)(Supplemental Fig. 2A), all of which can mediate autocrine activation. To assess the broader biological implications of FER loss, Reactome pathway analysis revealed significant enrichment of ‘Non-integrin membrane–ECM interactions’ (NES = 2.07, FDR = 0) and ‘MET promotes cell motility’ (NES = 1.89, FDR = 0.005) in FER-high cells (Fig. 2B and Supplemental Table 3). Next, we defined a cutoff based on the data distribution (|log2FC| ≥ 0.4 and adj. *p* < 0.001) to determine if the highly differentiated genes contribute to growth factor dependent pathways. Using this threshold, FER-high cells exhibited 46 genes significantly upregulated and 12 genes significantly downregulated when compared with FER-depleted cells (Supplemental Fig. 2B). Moreover, submission of the 46 upregulated genes to over-representation analysis (ORA) revealed enriched Molecular Function GO terms included ‘GFR ligand activity’ (adj. *p* = 2.13 × 10⁻⁴), ‘receptor ligand activity’ (adj. *p* = 3.87 × 10⁻⁴), ‘EGFR binding’ (adj. *p* = 1.23 × 10⁻³), and ‘GFR binding’ (adj. *p* = 1.99 × 10⁻³) (Supplemental Fig. 2C and Supplemental Table 4), and Reactome pathways included ‘RTK signaling’ (adj. *p* = 3.33 × 10⁻⁷), ‘Signal transduction’ (adj. *p* = 8.88 × 10⁻⁷), and ‘Signaling by MET’ (adj. *p* = 1.79 × 10⁻⁶) (Supplemental Fig. 2D and Supplemental Table 5). From these data, we selected and prioritized EGFR and MET; two GFRs that are clinically relevant in HNSCC [23,31]. To supplement the relevance of these findings, a combination of immunohistochemistry (IHC) and immunofluorescence (IF) showed that EGFR and MET are highly expressed in HNSCC cells at the invasive front, both in primary tumors and their derivative invasive HNSCC PDO models (Supplemental Fig. 2E).

To examine the potential FER-mediated interplay with GFRs, we next performed an IF for FER in combination with MET or EGFR in T4 or T8 PDO models expressing FER-iKD #1, henceforth referred to as FER-iKD, and observed that both GFRs are enriched in invasive structures, where they colocalize with FER (Fig. 2C and D). FER depletion resulted in a reduction in both GFR expression levels (Fig. 2C and D). Although western blotting also showed a reduction in GFR levels after FER knockdown (Fig. 2E), subsequent quantification of EGFR and MET using FACS showed that the EGFR and MET expression are not significantly reduced at the cell surface upon FER knockdown (Fig. 2F and G). In short, our data indicate that FER colocalizes with GFRs at the plasma membrane of invasive cells and suggest that FER may facilitates the activation of GFR pathways involved in invasive growth and metastasis of HNSCC.

### FER is required for GFR-induced invasive growth in HNSCC

**Fig. 3:**
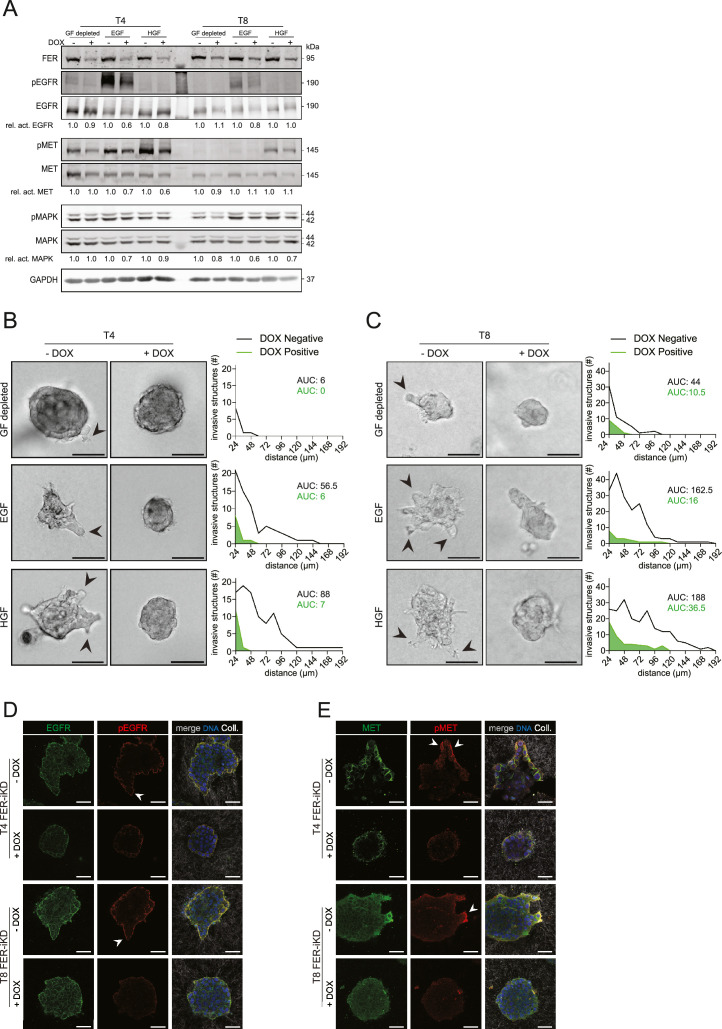
FER expression is essential for GFR-dependent HNSCC invasion A) FER regulates GFR signaling. HNSCC FER-iKD PDO models were cultured in the absence (-) or presence (+) of DOX and either starved or stimulated with EGF or HGF and analyzed by western blotting. Relative activation of EGFR, MET and MAPK (phosphorylation/total) are shown below the panels. GAPDH was used as loading control. B and C) Brightfield images show the effect of FER knockdown (+DOX) in T4 (B) and T8 (C) with or without EGF or HGF. Black arrowheads indicate invasive strands. Scale bars: 50 µm. Strand invasion from three independent experiments was quantified and displayed in line graphs adjacent to each brightfield image (*n* = 40 PDOs per condition). The area under the curve (AUC) was employed to quantify differences in strand length between conditions. D and E) The impact of FER loss on GFR expression and activation was analyzed for EGFR (green) and pEGFR (red, D), or MET (green) and pMET (red, E). The right panels show merged images including Collagen-I (white) and DNA (blue). Arrowheads (middle images) indicate phosphorylation in invasive strands. Scale bars: 50 µm.

To examine if FER controls GFR-dependent invasive growth, we first assessed the biochemical impact of FER loss on GFR activation. For this, PDOs were stimulated with EGF or HGF and analyzed for EGFR or MET activation on the respective Y1068 and Y1234/5 phosphorylation sites. FER knockdown resulted in approximately 40 % and 20 % reduction of EGFR phosphorylation levels in T4 and T8, respectively (Fig. 3A). The impaired activation of MET was evident in FER-depleted T4, but not in T8 (Fig. 3A). Importantly, phosphorylation of the GFR signaling effector MAPK was inhibited to a similar extent (approximately 30 % reduction) when stimulated with EGF or HGF in both models (Fig. 3A). The reciprocal experiment was performed using T4 FER reconstitution, showing that activation of both GFRs and their downstream effector MAPK, were rescued (Supplemental Fig. 3). Our data indicate that FER is essential for optimal activation of the GFRs EGFR and MET.

Next, we tested if the FER-dependent activation of EGFR and MET also impacts the EGF or HGF-dependent PDO invasion into Collagen-I. Both models displayed modest invasion when seeded in Collagen-I matrices in GF-depleted medium. PDO T8 showed substantial invasion under these conditions, indicating the presence of alternative and/or autocrine signaling cues, which were nonetheless effectively blocked upon FER loss (Fig. 3B and 3C, top panels). Stimulation with EGF or HGF resulted in a significant increase of invasive growth in both models, a phenotype that was critically dependent on the expression of FER (Fig. 3B and C, middle and bottom panels). We also investigated *in situ* GFR activation and observed that phosphorylation of MET and EGFR is largely restricted to the invasive cells, a spatial activation that is dependent on FER expression (Fig. 3D and E). In short, our data show that FER acts as a regulator of GF-dependent EGFR and MET activation and thereby imply that FER promotes the invasive growth of HNSCC.

### FER regulates the endosomal transport of pro-invasive GFR signals

**Fig. 4:**
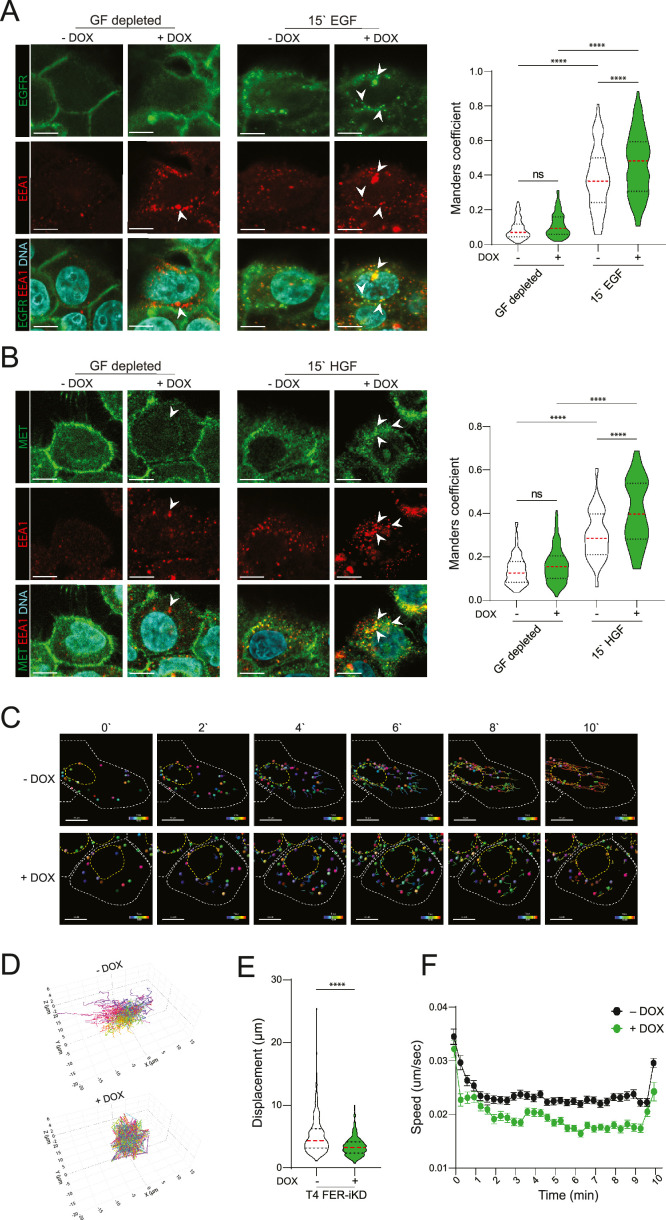
FER regulates endosomal trafficking of growth factor receptors in HNSCC A and B) FER depletion leads to the accumulation of MET and EGFR in early endosomes. T4 FER-iKD PDO models were starved or stimulated with EGF or HGF in the absence or presence of DOX and immunofluorescence probed for EEA1 (red) and either EGFR (green, A) or MET (green, B). Merged images including DNA signals (blue) are shown in the bottom panels. Scale bars: 15 µm. Violin plots show the co-occurrence of EEA1 (*n* = 150 spots per condition) with EGFR or MET using Manders` coefficients from three independent experiments. C and D) EGF cellular uptake (as a GFR intracellular trafficking proxy) was tracked in the T4 FER-iKD HNSCC PDO in the absence or presence of DOX. Scale bars: 10 µm (C). Alexa-555 conjugated EGF signals are plotted for both conditions in a 3-dimensional trajectory plot in (D). E and F) Fluorescent EGF total track displacement (E) and spot speed (F) were quantified for each condition from three independent experiments (*n* = 130 spots per condition). Error bars indicate SD; ns indicates non-significant, ****p < 0.001.

Because FER loss attenuates GF dependent activation of EGFR and MET but does not significantly reduce GFR expression levels at the plasma membrane, we analyzed if endosomal transport could underlie the observed FER dependent GFR activation. For this we stimulated PDOs with either HGF or EGF in the context of FER loss and analyzed localization of EGFR or MET in conjunction with the early endosomal marker EEA1. We first assessed the role of FER on endosomal formation and observed that FER-iKD in growth factor depleted HNSCC PDO cells leads to a significant increase in EEA1 signal intensity and signal accumulation, indicative of impaired recycling due to an accumulation of early endosomal vesicles in both models (Supplemental Fig. 4). We subsequently focused our efforts on the T4 model, as this PDO model does not exhibit overt autocrine pro-invasive pathway activation (see Fig. 3B). Stimulation with either EGF or HGF induced a marked increase in the colocalization of EEA1 with EGFR and MET in the FER-depleted T4 PDO (Fig. 4A and B).

To better understand how FER-dependent endosomal trafficking affects GFR dynamics, we analyzed the endocytic uptake of EGF. For this, we incubated T4 FER-iKD cells with fluorophore-conjugated EGF in the absence or presence of DOX and imaged endosomal formation and dynamics in real-time. In control cells, EGF-containing endosomes showed distinct trajectories towards the perinuclear region, which were accompanied by high intracellular displacement and speed (Fig. 4C – 4F and Supplemental Videos 1 and 2). In contrast, FER-depleted cells showed aberrant localization and reduced velocity of the EGF-containing vesicles (Fig. 4C – F and Supplemental Videos 1 and 2). Overall, our findings thereby suggest that the activation of GFR signals and the subsequent invasive growth of HNSCC are, in part, controlled by a FER-dependent modulation of endosomal trafficking.

### FER is essential for invasive growth and metastasis

**Fig. 5:**
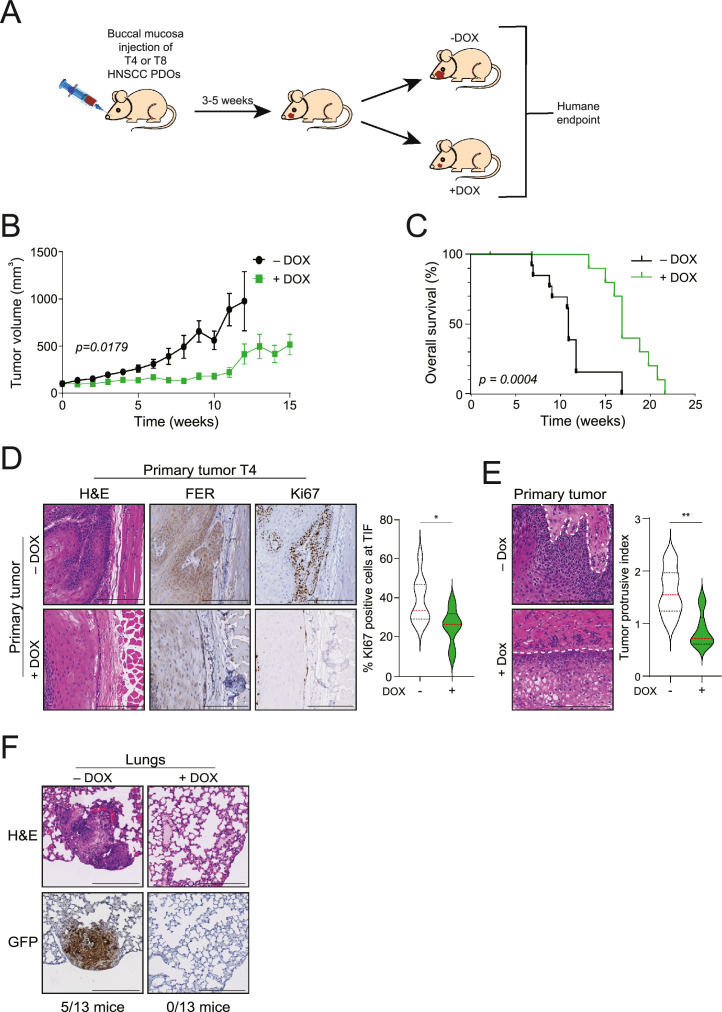
FER controls invasive growth and metastasis of HNSCC *in vivo* A) T4 or T8 FER-iKD cells were transplanted into the buccal mucosa of recipient mice. Upon the development of palpable tumors, half of the mice were switched to a DOX-containing diet to induce FER knockdown. B and C) FER controls tumor growth and survival. T4 PDX tumor volumes (B) were measured weekly using digital calipers for both the control (- DOX) and FER knockdown (+ DOX). Error bars = s.e.m, *n* = 13. The Kaplan–Meier overall survival plot from the mice in (B) are shown in (C). D) Representative images of the tumor invasive front in FER-iKD mice. Shown are tissue sections stained for hematoxylin and eosin (H&E), FER (middle panels), or Ki67 (right panels). Scale Bars: 200 µm. The violin plot shows the quantification of Ki67-positive cells at the tumor-invasive front. E) H&E primary tissue sections depicting the effect of FER loss on invasive patterns into the surrounding stroma. The white outline indicates the tumor boundary. Scale Bars: 200 µm. The violin plot shows the quantification of tumor protrusive indexes. F) Representative lung sections from control and FER knockdown mice were analyzed for the presence of tumor cells using immunohistochemistry for GFP. Scale Bars: 200 µm. Text below brightfield images indicate the number of mice with metastasis to the lungs. Error bars indicate SD; *p < 0.05, **p < 0.01.

Since our data indicate that FER kinase controls the activation of multiple GFRs, we asked if invasive growth *in vivo* also depends on FER function. T4 or T8 PDOs were transplanted into the buccal mucosa of recipient male mice (PDX) and placed on either control food or a DOX-containing containing diet to induce FER knockdown after tumor volumes reached 50 mm^3^ (Fig. 5A). Depleting FER in the T4 PDX model led to a significant reduction in tumor growth over time (Fig. 5B) and a corresponding increase in overall survival (Fig. 5C). The T8 PDX model did not show a reduction in tumor volumes or an effect on survival (Supplemental Fig. 5A and 5B), suggesting that alternative mechanisms omit FER depletions (also see Fig. 3C and Supplemental Fig. 1D). Upon histological examination of the T4 primary tumors, we noted a decrease in FER expression levels and a reduction in proliferating cells, with observable changes in cellular differentiation (Fig. 5D). Furthermore, there was a significant reduction in local tumor invasion at the invasive front in the FER knockdown cohort in both the T4 and T8 PDX models (Fig. 5E and Supplemental Fig. 5C). Importantly, FER loss fully prevented the development of metastasis to the lungs (T4 control PDX: 5/13 mice; T8 control PDX: 1/13 mice) (Fig. 5F and Supplemental Fig. 5D), which underscores our hypothesis and the invasion data using 3D Collagen-I gels. In short, these results substantiate our hypothesis that FER controls HNSCC invasion *in vivo* and suggest that FER promotes non-selective GFR activation.

## Preclinical proof of concept: a FER PROTAC intervention to treat invasive HNSCC

Since targeting either EGFR or MET does not provide significant prognostic benefit for HNSCC patients [24,[34], [35], [36], [37]], we tested the assumption that single-agent targeted inhibition of GFRs is not effective due to redundant oncogenic GFR activity mediated by FER. For this, we started by testing the effect of approved pharmacological GFR inhibitors targeting EGFR or MET [38,39]. Biochemical assessment confirmed that Afatinib or Capmatinib treatment impairs their respective GFR target and downstream activation of MAPK, and that dual inhibition of EGFR and MET is required to abrogate EGFR, MET, and downstream MAPK activity (Supplemental Fig. 6A). Importantly, targeted inhibition of EGFR and/or MET does not affect FER expression (Supplemental Fig. 6A), suggesting that FER may sustain invasive tumor growth independently of specific GFR antagonism. Next, we investigated if high FER expressing PDOs can facilitate redundant GFR-mediated invasion as a mechanism for therapeutic resistance to Afatinib or Capmatinib treatment with opposing administration of HGF or EGF in T4 or T8 high FER conditions. Indeed, HGF could restore invasion in the presence of EGF and Afatinib (Supplemental Fig. 6B). Similarly, EGF rescued invasion in Capmatinib-treated cells that were stimulated with HGF (Supplemental Fig. 6C). As expected, simultaneous inhibition of EGFR and MET with Afatinib and Capmatinib in the presence of EGF and HGF, results in an almost complete cessation of tumor cell invasion in 3D (Supplemental Fig. 6D). Together, these data demonstrate functional redundancy in GFR activation during invasive HNSCC growth and suggest that FER may facilitate activation of multiple GFR pathways, contributing to therapeutic resistance to GFR inhibitors.

**Fig. 6:**
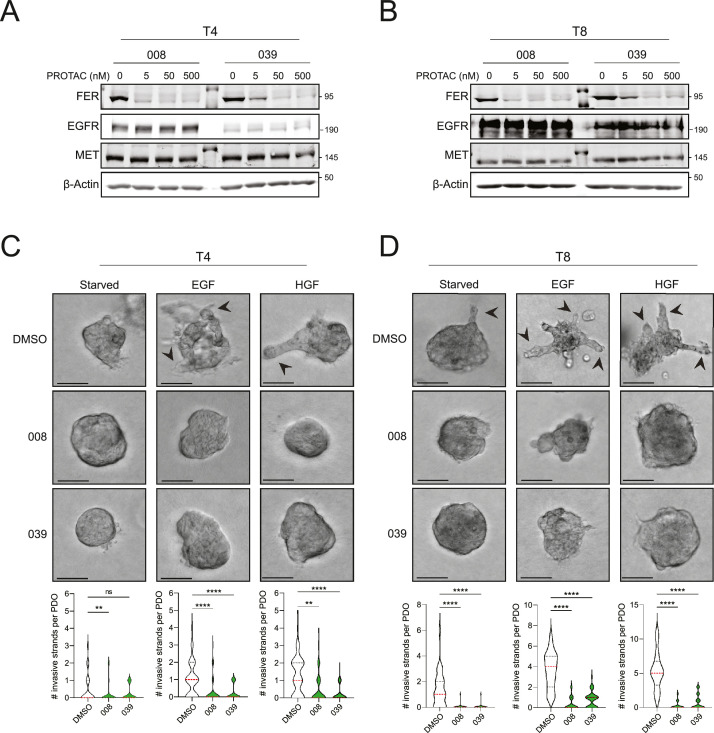
FER-targeting PROTAC compounds suppress growth factor dependent invasion in HNSCC PDOs A and B) Western blot analysis of T4 (A) or T8 (B) PDOs cultured in the absence or presence of the FER PROTAC compounds, SIAIS352008 (008) or SIAIS262039 (039). Blots were probed for FER, EGFR and MET. β-actin was used as loading control. Note the specific degradation of FER. C and D) FER-targeting PROTACs impair EGF and HGF-dependent invasion in HNSCC cells. T4 (C) or T8 (D) HNSCC PDO models were cultured in a Collagen-I matrix to allow invasive growth. HGF or EGF was added in the presence or absence of 50 nM 008 or 039 and assessed for invasion (DIC images). Arrowheads (black) indicate invasive strands. The number of invasive strands per PDO (*n* = 40 per condition) was quantified from three independent experiments (bottom violin plots). Error bars indicate SD; ns indicates non-significant, **p < 0.01, ****p < 0.001.

Since our data suggest that FER is responsible for unsuccessful GFR-targeted therapy due to redundant receptor activation, we employed a FER-specific PROteolysis-Targeting Chimera (PROTAC) approach as a therapeutic option to treat invasive HNSCC [40]. As a proof of concept, we started testing the efficacy of two FER-specific PROTAC compounds SIAIS352008 (008) and SIAIS262039 (039) on the T4 and T8 PD models. Both compounds induced a dose-dependent reduction of FER expression, showing near-complete inhibition at 5 nM for 008 and 50 nM for 039, respectively (Fig. 6A and B). Additionally, neither 008 nor 039 caused a reduction in MET or EGFR expression (Fig. 6A and B). To test the functional antagonism of the FER PROTACs, EGF or HGF-stimulated invasion assays were performed on all HNSCC PDO models (T1, T4, T5 and T8), which showed that both 008 and 039 effectively prevented GFR-dependent invasive growth (Fig. 6C and 6D and Supplemental Fig. 7A and 7B).

**Fig. 7:**
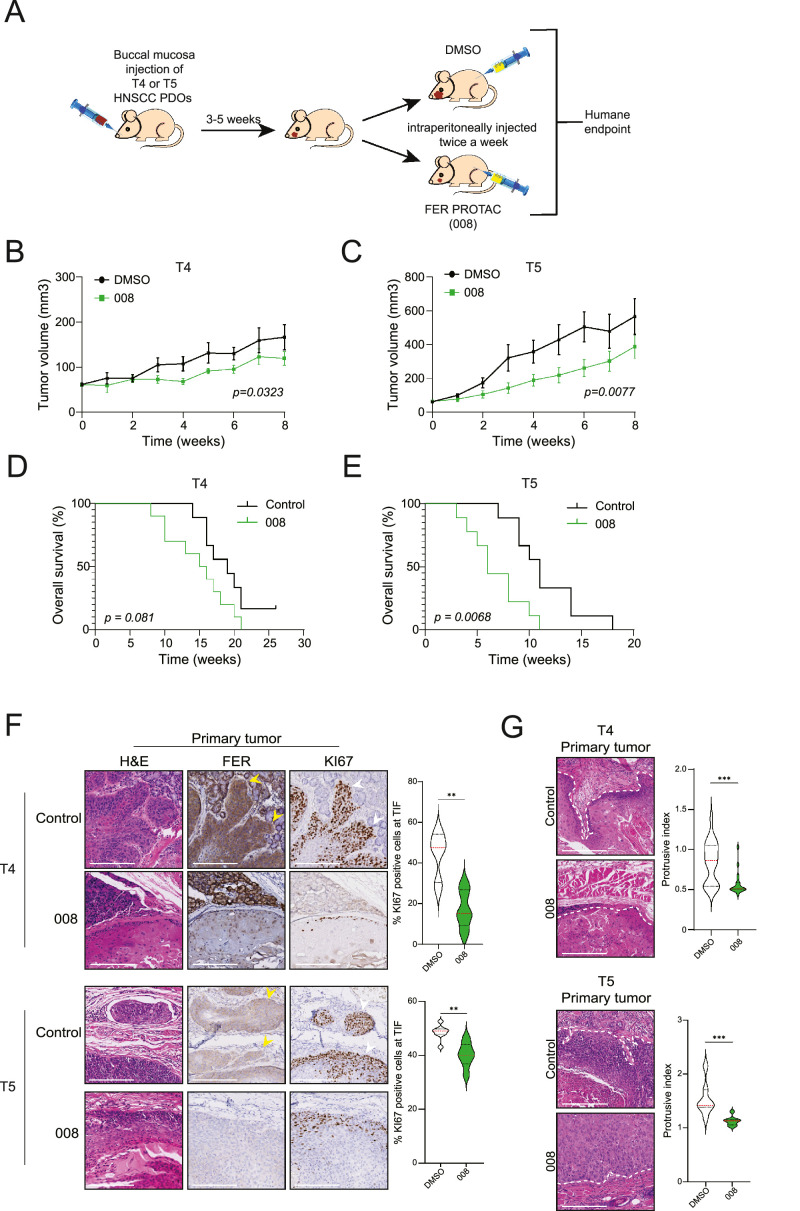
Preclinical targeting of HNSCC using FER-specific PROTACs A) T4 and T5 PDO cells were transplanted into the buccal mucosa of recipient mice. Upon the development of palpable tumors, half of the mice were intraperitoneally injected twice a week with DMSO (control, sham) or a FER-targeting PROTAC (compound 008). B and C) T4 and T5 HNSCC PDX tumor volumes were measured weekly using digital calipers. Error bars = s.e.m., *n* = 13 for both the control and 008 treated mice. D and E) Kaplan–Meier survival plot of the mice shown for T4 in (B) and T5 in (C). F) Representative sections of the tumor invasive front of T4 (upper panels) and T5 (lower panels) primary tissue sections from control or PROTAC treated mice were analyzed for tumor histology (H&E), FER expression (middle panels) and Ki67 (right panels). Scale Bars: 200 µm. Yellow and white arrowheads indicate high FER and KI67 expressing cells at the tumor-invasive front, respectively. The violin plot shows the quantification of Ki67 cells at the tumor-invasive front of control and PROTAC-treated mice. G) Representative sections from T4 (upper panels) and T5 (lower panels) primary tumors, depicting invasive patterns into the surrounding stroma in a control (top) and 008 treated mouse (bottom). The white dashed line indicates the tumor boundary. Scale bars: 200 µm. The violin plot shows the quantifications of the tumor protrusive indexes. Error bars indicate s.e.m; ns indicates non-significant, **p < 0.01, ***p < 0.005, ****p < 0.001.

We next transplanted T4 and T5 PDO models into the buccal mucosa of recipient male mice and treated them intraperitoneally with either DMSO or compound 008 when tumors reached a volume of >50 mm^3^ (Fig. 7A). T1 was excluded from in vivo studies due to poor outgrowth. Treatment using compound 008 (1 mg/kg) showed a significant reduction in longitudinal tumor volume and survival in the T4 and T5 PDX models (Fig. 7B – 7E). As expected, both PDX models showed reduced FER expression and low numbers of proliferating cells in the presence of PROTAC 008 (Fig. 7F). Depletion of FER using 008 markedly impaired invasive growth patterns in the T4 and T5 primary tumors, resulting in circumscribed tumor margins with signs of epidermal differentiation (Fig. 7G). We did not detect metastasis to the lungs after 8 weeks of treatment in both control and treated mice. Taken together, our data show that FER regulates invasive growth, and that successful preclinical intervention in mice using PROTACs provides a successful proof of concept for FER as a promising therapeutic option in HNSCC.

## Discussion

Mechanisms by which cancer cells overcome oncogene addiction in the context of GFR inhibition have undergone extensive investigations [[41], [42], [43], [44]] and it is well-recognized that redundant GFRs can undermine inhibition strategies [[45], [46], [47], [48]]. Clinical and preclinical data indeed show that single or dual-agent antagonism against EGFR and/or MET receptor does not prevent tumor progression [24,34,[49], [50], [51]]. In the current manuscript, we have identified the nonreceptor tyrosine kinase FER as a regulator of oncogene addiction in HNSCC. We provide mechanistic insight into the control over GFRs and demonstrate that FER represents a clinically prognostic and targetable regulator of GFR activation in cancer cells.

GFRs can transactivate multiple other tyrosine kinase receptors as a mechanism of therapeutic resistance. In particular, MET has been shown to transactivate ERBB1/EGFR, ERBB2, ERBB3, AXL, RON and PDGFR-α [29,[52], [53], [54], [55]]. Similarly, EGFR can transactivate RON [56] and transactivate IGF1R through heterodimerization, promoting cetuximab resistance in HNSCC [57]. Furthermore, several studies have shown that GFRs form complexes with integrins to mediate cell-ECM adhesion during invasion [[58], [59], [60], [61]]. Together, this illustrates the detrimental effect of receptor usage and receptor promiscuity in cancer. Since FER regulates the recycling of ECM-dependent adhesion complexes in cancer [5,62,63], it indicated that FER may also control GFR activation during invasive growth.

Indeed, our data now indicate that FER controls indiscriminate propagation of GFR signaling, and as such, is an upstream facilitator of GFR-induced activity. Interestingly, we have not observed a sustained reduction in cell surface expression levels of either MET or EGFR upon loss of FER. The binding of ligands to GFRs is rapidly pursued by endocytosis, followed by persistent and regulated GFR activation in early endosomal compartments and rapid recycling back to the cell surface or lysosomal degradation [18,[64], [65], [66]]. Our data indicate that FER is essential for the early stages of ligand-mediated autophosphorylation of the EGFR and MET receptors on the residues Y1068 and Y1234/5 respectively. However, we also demonstrate that FER is necessary for correct intracellular transport and processing of internalized EGF ligands. It therefore remains unclear which exact mechanisms control the propagation of the FER-dependent GFR signals during endosomal recycling. A possible explanation is the finding that FER can function as a sensor of high membrane curvature to regulate nascent tubule elongation during endocytosis to promote its kinase activity [11,67]. As such, it is plausable that initial phosphorylation events instigate plasma membrane curvature that induces recruitment of FER and other effectors, and that this recruitment is essential for the potentiation and propagation of GFR activation during endosomal trafficking and recycling. Supporting such a scenario are findings that FER promotes rapid recycling of the α6 and β1 integrin receptors [12] and EGFR recycling through phosphorylation of PKCδ [13]. While future work will have to further dissect the exact underlying molecular mechanisms and their impact on different invasive carcinoma types, it appears that FER plays a bifunctional role in both the activation and redistribution of cell-ECM adhesion receptors and GFRs back to the plasma membrane during tumor invasion. Supporting this are our data demonstrating that FER is essential for the intracellular displacement, directionality, and speed of EGF-containing endosomes. Together with the effect of FER on GFR activation, this suggests that persistent endosomal-dependent GFR signaling is modulated or promoted by FER. Indeed, previous reports showed that EGFR-mediated MAPK activation at the plasma membrane leads to a slow and consistent build-up of nuclear phosphorylated (p)MAPK, whereas EGFR-endosomal activation results in a rapid yet brief pMAPK activity in the nucleus [20]. Similarly, phosphorylated MET receptors can accumulate in peripheral endosomes to sustain MAPK activity through PKCε during migration, or localize to perinuclear endosomal compartments to augment STAT3 activity [18,68]. Because STAT3 is activated upon FER expression [69] and MAPK1 has been identified as a direct FER substrate [12], FER is likely a multifactorial driver of invasive cancer growth.

Our data also indicate residual GFR activation and invasion in GF-depleted conditions, suggesting potential autocrine or alternative modes of activation. Indeed, mRNA sequencing data pointed to a scenario where in the absence of *EGF* or *HGF* expression, other EGFR activating ligands like transforming growth factor alpha (*TGFA*), Heparin-binding EGF-like growth factor (*HBEGF*), Epiregulin (*EREG*) and Amphiregulin (*AREG*), were transcriptionally elevated in FER high conditions. Because these factors have been described as mediators of invasion [[70], [71], [72], [73]], they are likely candidates for the tumor cell-intrinsic control of GFR-driven invasive growth in these models. This was particularly evident in PDO T8, which is why we have omitted this model in the preclinical PROTAC experiments. Notably, recent work has shown that HNSCC exhibits distinct combinations of EGFR ligand expression such as TGFA, HBEGF, EREG, and AREG, which define highly active EGFR states; in particular, AREG- and EREG-driven subtypes are associated with EMT and local invasion [74]. Interestingly, cancer-associated fibroblasts (CAFs) engage in bi-directional AREG-EGFR signaling with partial EMT of HNSCC cells to induce cancer cell invasion [75], indicating a potential role for FER in mediating cancer cell–host communication. Furthermore, elevated AREG expression associates with tumor budding, invasion, poor prognosis, and increased sensitivity to EGFR inhibition in HNSCC, while TGFα can confer cetuximab resistance through inducing EGFR-MET interaction in colon cancer [[76], [77], [78]]. Moreover, previous reports have suggested that FER might activate MET and EGFR independent of exogenous ligands [79,80], and that activation of MET signaling can induce resistance towards VEGFR inhibitors [81]. Combined, these findings imply that promiscuous use of GFR ligands propels and fosters the FER-dependent oncogene addiction towards GFR activation. Given that we observed downregulation of *VEGF* and *PDGF* ligands upon loss of FER expression, we envision that FER may promote a feed-forward loop through transcriptional activation of EGFR and other GFR ligands as a mechanism to drive invasive growth. While we have no formal evidence that VEGFR and PDGFR control invasive growth in our models, it is well-established that these receptors are important drivers of tumor progression in HNSCC [82,83].

In summary, we have identified FER as a regulator of HNSCC invasive growth through non-discriminate and endosome-dependent activation of GFR signaling. Given this, and the clinical challenges in the management of HNSCC based on GFR inhibitors, we have employed a FER-specific PROTAC-based approach. Our preclinical genetic and pharmacological intervention studies in primary patient organoids and PDX models of HNSCC, combined with clinical prognostic association studies, provide an encouraging platform to counter therapy resistance against GFR inhibitors by targeting of FER kinase. We anticipate that a FER-based treatment will not only be beneficial in HNSCC but may also extend towards other basal-type invasive carcinomas such as triple negative breast cancer (TNBC).

## Methods

Full details on the materials and methods are described in *SI Appendix*. HNSCC patient-derived organoids (PDOs) were cultured and seeded in Collagen-I as previously described [84,85]. FER knockdown (FER-iKD #1 or FER-iKD #2) or full-length and RNAi-resistant reconstitution (FER::Recon) were generated using a Lentiviral doxycycline (DOX)-inducible system as described previously [12,86]. Protein expression was determined using Western blotting. Single cell mRNA sequencing was performed by Single Cell Discoveries and visualized using Cellenics. Formalin-Fixed Paraffin-Embedded (FFPE) tissue sections were immunohistochemically stained and scored. Immunofluorescence imaging was used to assess protein spatial expression, and colocalization analysis was performed using CellProfiler [87]. Live cell imaging of conjugated EGF was conducted and quantified using IMARIS. Cell surface protein expression was evaluated using flow cytometry. Invasion assays were performed as previously described [85] and quantified using Fiji. PDOs were orthotopically transplanted into male mice and assessed for local invasion, proliferation and metastasis.

## Data availability

Single-cell mRNA sequencing data reported in this paper have been deposited in the NCBI Gene Expression Omnibus (GEO) database (RRID:SCR_005012), www.ncbi.nlm.nih.gov/geo (accession no GSE255878).

## CRediT authorship contribution statement

**Peter D. Haughton:** Writing – review & editing, Writing – original draft, Visualization, Validation, Software, Methodology, Investigation, Formal analysis, Data curation, Conceptualization. **Lotte N.F.L. Enserink:** Methodology, Data curation. **Sandra Tavares:** Investigation. **Wisse Haakma:** Data curation. **Garik Galustjan:** Data curation. **Sjors Koppes:** Formal analysis, Data curation. **Lorenza Casasanta:** Formal analysis. **Else Driehuis:** Resources. **Hans Clevers:** Resources. **Yanchun Zhang:** Resources. **Gaofeng Fan:** Resources. **Stefan Willems:** Investigation. **Xiaobao Yang:** Visualization, Resources, Methodology, Investigation. **Patrick W.B. Derksen:** Writing – review & editing, Visualization, Supervision, Investigation, Funding acquisition, Conceptualization.

## Declaration of competing interest

The authors declare that they have no known competing financial interests or personal relationships that could have appeared to influence the work reported in this paper.

## Appendix Supplementary materials

Image, application 1

Image, application 2

Image, application 3

Image, application 4

Image, application 5

Image, application 6

Image, video 7

Image, video 8
