# Supplementary material for: FER kinase governs invasive growth of head and neck squamous cell carcinoma through dynamic control of growth factor receptor activity
Source: Neoplasia. 2025 Oct 19;70:101241. doi: 10.1016/j.neo.2025.101241 (PMC12569816; doi:10.1016/j.neo.2025.101241)
Supplement: Supplementary file 1 [file mmc1.docx]

Supporting information for

FER Kinase governs Invasive Growth of Head and Neck Squamous Cell Carcinoma through dynamic control of Growth Factor Receptor activity

Peter D. Haughton^1^, Lotte N.F.L. Enserink^1^, Sandra Tavares^1^, Wisse Haakma^1^, Garik Galustjan^1^, Sjors Koppes^1^, Lorenza Casasanta^1^, Else Driehuis^2^, Hans Clevers^2,3^, Yanchun Zhang^4^, Gaofeng Fan^4^, Stefan Willems^5^, Xiaobao Yang^6^* and Patrick W.B. Derksen^1^*

*Correspondence:

Patrick WB Derksen, [p.w.b.derksen@umcutrecht.nl](mailto:p.w.b.derksen@umcutrecht.nl)

Xiaobao Yang, [yang.xiaobao@gluetacs.com](mailto:yang.xiaobao@gluetacs.com)

This PDF file includes:

Supplementary Materials and Methods

Figures S1 to S8

Video legends S1 and S2

**Materials and Methods**

*Organoid culture*

HNSCC patient-derived organoids (PDO); T1 (tongue squamous cell carcinoma), T4 (tongue squamous cell carcinoma), T5 (parotid gland-localized SCC), and T8 (gingiva SCC) were cultured as previously described (1). Briefly, PDOs were cultured in Basement Membrane Extract (BME, R&D Systems, Cat# 3533-005-02) and passaged using trypsin/EDTA (T3924, Sigma), 37 °C for 10 minutes. 10 mL of ice-cold PBS was added and cells were centrifuged for 5 minutes at 1,500 rpm. Cells were subsequently seeded in 40 µL drops of BME and grown in Advanced DMEM/F12 media (Gibco) supplemented with 1% Pen/Strep (Lonza), 1% HEPES (Gibco), 1% glutamax (Gibco), 1 × B27 supplement (Life Technologies, Cat# 17504-044), 1.25 mmol/L N-acetyl-l-cysteine (Sigma-Aldrich, Cat# A9165), 10 mmol/L Nicotinamide (Sigma-Aldrich, Cat# N0636), 50 ng/mL human EGF (PeproTech, Cat# AF-100-15), 500 nmol/L A83-01, 10 ng/mL human FGF10 (PeproTech, Cat# 100-26), 5 ng/mL human FGF2 (PeproTech, Cat# 100-18B), 1 μmol/L Prostaglandin E2 (Tocris Bioscience, Cat# 2296), 0.3 μmol/L CHIR 99021 (Sigma-Aldrich, Cat# SML1046), 1 μmol/L Forskolin [Bio-Techne (R&D Systems) Cat# 1099], 4% R-spondin, and 4% Noggin (both produced via the r-PEX protein expression platform at U-Protein Express BV). The media was refreshed every 3 days. PDOs were cultured at 37°C in 5% CO_2_ conditions.

For growth factor inhibition and stimulation experiments, PDOs were starved in Advanced DMEM F-12 medium (Thermo Fisher Scientific) containing 1% Pen/Strep and 1% HEPES buffer (Thermofisher Scientific) for 5 days. For invasion assays, growth factors (200 ng/mL HGF (Peprotech) or 200 ng/mL EGF (Thermo Fisher Scientific) and the specified inhibitors (1 μM Afatinib (Selleckchem, Cat# S1011) or 100 nM Capmatinib (Selleckchem, Cat# S2788)) or PROTACs (50 nM SIAIS352008 (008) or SIAIS262039 (039)) were added for 3 days prior to fixation. For western blot, samples were incubated with inhibitors for 1 hour or PROTACs for 6 hours and stimulated with growth factors for 15 minutes, prior to lysate preparation.

*Antibodies and reagents*

The following primary antibodies were used for immunofluorescence and immunohistochemistry: rabbit anti-Keratin 14 (1:200, Cat# 905301, Biolegend, RRID:AB_2565048), rabbit anti-FER (1:100, Cat# NBP1-20089, Novus Biologicals), mouse anti-FER (1:100, Cat# 4268S, Cell Signaling Technologies, RRID:AB_1642037), rabbit anti-EGFR (1:100, Cat# 4267, Cell Signaling, RRID:AB_2246311), rabbit anti-phospho-EGFR (Tyr1068) (1:200, Cat# 3777, Cell Signaling, RRID:AB_2096270), rabbit anti-MET (1:200, Cat# 8198, Cell Signaling, RRID:AB_10858224), rabbit anti-phospho-MET (Tyr1234/1235) (1:100, Cat# 3077, Cell Signaling, RRID:AB_2143884), rabbit anti-EEA1 (1:100, Cat# 3288, Cell Signaling, RRID:AB_2096811) and mouse anti-P63 (1:100, Cat# M7247, Agilent, RRID:AB_2207187). The Roche Ventana™ was used to stain Ki67 (Cat# 790-4286, Ventana Medical Systems, RRID:AB_2631262). The following secondary antibodies were used: Alexa-fluor-488/568/647-conjugated goat anti-mouse (1:500, Thermo Fisher Scientific:  Cat# A-11001 (RRID:AB_2534069), Cat# A-11004 (RRID:AB_2534072), Cat# A-21235, RRID:AB_2535804), respectively) or goat anti-rabbit antibodies (1:500, Thermo Fisher Scientific: Cat# A32731 (RRID:AB_2633280), Cat# A-11011 (RRID:AB_143157), Cat# A-21245 (RRID:AB_2535813), respectively). To visualize DNA and F-actin, 4′,6-diamidino-2-phenylindole (DAPI, Thermo Fisher), and Alexa-fluor-568-conjugated phalloidin (Thermo Fisher) were used, respectively.

The following antibodies were used for western blot: rabbit anti-phospho-p44/42 MAPK (1:1000, Cat# 9101s, Cell Signaling, RRID:AB_331646), rabbit anti-GAPDH (1:10,000, Cat# Mab374, Millipore, RRID:AB_2107445), mouse anti-AKT (1:1,000, Cat# 2920, Cell Signaling, RRID:AB_1147620), mouse anti-β-actin (1:1,000, Cat# NB600-501, Novus Biologicals, RRID:AB_10077656), rabbit anti-V5 (1:1,000, Cat# 13202, Cell Signaling, RRID:AB_2687461), mouse anti-FER (1:1,000, Cat# 4268S, Cell Signaling, RRID:AB_2278286), mouse anti-p44/42 MAPK (1:1000, Cat# 9107, Cell Signaling,  RRID:AB_10695739), rabbit anti-EGFR (1:1,000, Cat# 4267, Cell Signaling, RRID:AB_2246311), rabbit anti-phospho-EGFR (Tyr1068) (1:1,000, Cat# 3777, Cell Signaling, RRID:AB_2096270), rabbit anti-phospho-MET (Tyr1234/1235) (1:1,000, Cat# 3077, Cell Signaling, RRID:AB_2143884) and mouse anti-MET (1:500, Cat# 187366, Thermofisher, RRID:AB_2533047). Secondary antibodies used for detection were: Alexa-fluor-680 goat anti-mouse (1:5,000; Thermofisher Scientific; Cat# A-21057 (RRID:AB_2535723)), Alexa-fluor-680 goat anti-rabbit (1:5,000; Thermofisher Scientific; Cat# A-21076 (RRID:AB_2535736)), Alexa-fluor-800 goat anti-mouse (1:5,000; Thermofisher Scientific; Cat# SA5-10176 (RRID:AB_2556756)) or Alexa-fluor-800 goat anti-rabbit (1:5,000; Thermofisher Scientific; Cat# SA5-10036 (RRID:AB_2556616). The following antibodies were used for Flow cytometry: PE/Cyanine7 conjugated human anti-EGFR (1:100, Cat# 352910, BioLegend, RRID:AB_2562159), PE/Cy5.5 conjugated human anti-MET (1:100, NBP2-50172PECY55, Novus Biologicals, RRID:AB_3322667)

*Lentiviral transduction*

Two independent doxycycline (DOX)-inducible shRNA constructs inducing FER knockdown (FER-iKD #1 or FER-iKD #2) and the full-length and RNAi-resistant reconstitution (FER::Recon) were generated as previously described (2, 3). Transduced FER-iKD samples were sorted for high GFP expression using FACS. To induce FER-KD or reconstitution, samples were treated with 1 μg/mL DOX (Cat# D9891, Sigma-Aldrich) for at least 3 days prior to experiments.

*Protein extraction and Western blot*

Sample buffer was added to PDO lysates, boiled at 95°C for 10 minutes and loaded in 8% - 10% SDS-PAGE gels for electrophoresis. Samples were transferred onto PVDF membranes, which were blocked with 5% w/v bovine serum albumin (BSA) (Merck & Co.; Rahway, New Jersey) in 1x TBS for 1 hour at room temperature. Subsequently, membranes were incubated with primary antibodies overnight at 4°C and washed four times with 1x TBST (Tween; Cat# 8.22184.0500, VWR International). Membranes were incubated with secondary antibodies in 1x TBST for 1 hour at room temperature, washed again, and imaged utilizing an Amersham Typhoon (Cytiva; Marlborough, Massachusetts, USA). Protein levels were quantified with Image Studio Lite software (LI-COR Biosciences; Lincoln, Nebraska, USA).

*Immunohistochemistry and Immunofluorescence on tissue sections*

Tissue sections (4 µm) were cut from paraffin-embedded samples and deparaffinized. Peroxidases were blocked for 15 minutes with 1.5% hydrogen peroxide. Antigen retrieval was carried out by boiling for 20 minutes in a pH 9.0 Tris-EDTA buffer, after cooling the sections were blocked with 1% BSA for 30 minutes. Primary antibody incubation was performed for 1 hour at room temperature, followed by washing the sections with PBS and incubating them with the Brightvision goat anti-rabbit HRP secondary antibody (Cat# Cat# DPVR110HRP, Immunologic, RRID:AB_2915958). After washing, the sections were stained for 10 minutes with BrightDAB (Cat# BS04, Immunologic), counterstained with hematoxylin for 20 seconds and coverslipped.

The hydrogen peroxide blocking step was not carried out for immunofluorescence on tissue sections. Secondary antibody incubations were performed with the previously mentioned Alexa-fluor conjugated antibodies at 1:200 dilution for 1 hour, and BrightDAB staining was replaced with a DAPI stain for 15 minutes.

*Patient-Derived Organoid (PDO) immunofluorescence*

PDO models were cultured in Collagen-I matrices (pre-polymerized at 26°C), formalin-fixed with 4% paraformaldehyde (PFA; Sigma-Aldrich) for 10 minutes at room temperature (20°C) and washed 3 x with 1x PBS. Samples were blocked with 10% normal goat serum (Gibco) in 0.3% Triton-X (Sigma-Aldrich) in 1x PBS for 1 hour. Primary antibodies were diluted in antibody buffer (0.3% Triton-X with 1% w/v BSA in 1x PBS) and incubated overnight at 4°C with shaking. Next, samples were washed 4 times with 1x PBS for 10-15 minutes and incubated with secondary Alexa-fluor-488/546/647-conjugated antibodies (1:500) together with DAPI (1 µg/mL) in antibody buffer at 4°C with shaking overnight. Samples were imaged using Zeiss LSM 880.

*Live fluorescence imaging and colocalization assessment*

T4 FER-iKD PDOs were cultured in BME supplemented with growth media for 3 days. PDOs were starved for 3 days and then half of the samples switched to starvation media containing DOX (1 mg/mL) for a further 2 days. Next, PDOs were treated with trypsin, spun down at 1,500 RPM at 4 °C, and 1x10E^4^ cells were seeded on Collagen-I coated 8-well glass-bottom plates (Ibidi, Gräfelfing, Germany). Cells were incubated with starvation media in the presence or absence of DOX for a further 24 hours. For live imaging, cells were stimulated with 200 ng/mL Alexa-555 conjugated EGF and imaged for 10 minutes using a Nikon spinning disk-based confocal microscope equipped with Plan Apo VC 100x N. A. At least 130 spots were tracked per condition using the IMARIS spot tracking software (version Imaris 10.1, RRID:SCR_007370). For colocalization experiments, cells were either starved or stimulated before fixation. Cells were labeled for rabbit anti-EEA1 (1:100, Cat# 3288, Cell Signaling, RRID:AB_2096811) and antibodies against rabbit anti-EGFR (1:100, Cat# 4267, Cell Signaling, RRID:AB_2246311) or rabbit anti-MET (1:200, Cat# 8198, Cell Signaling, RRID:AB_10858224) and imaged using the Zeiss LSM 880. The degree of colocalization was assessed using CellProfiler (4). Manders colocalization coefficient was used to determine the fraction of GFR that colocalized with EEA1.

*Flow Cytometry*

PDOs were cultured for 5 days, treated with trypsin, spun down at 1,500 rpm at 4 °C, and 1X10E^6^ cells were placed in Eppendorf tubes. The conjugated primary antibody was incubated for 30 minutes on ice in a 1% BSA solution. Samples were washed in ice-cold PBS, and centrifuged at 4 °C for 5 minutes, and the supernatant was removed. Samples were resuspended in 1% BSA PBS solution and fluorescence signals were quantified using a FACSCelesta flow cytometer, RRID:SCR_019597 (BD Biosciences; Franklin Lakes, New Jersey, USA), and analyzed with Flowing Software 2.5.1, RRID:SCR_015781 (Turku Bioscience; Turku, Finland). 1 µg/mL DAPI was added to the samples directly prior to the fluorescence analysis to exclude dead cells.

*Collagen invasion assay*

Invasion assays were performed as previously described (5).

*Invasion assay quantification*

Organoid invasion was analyzed using Fiji ImageJ2 2.9.0 software (RRID:SCR_002285) with the addition of a Concentric Circles plugin (Wayne Rasband; <https://imagej.nih.gov/ij/plugins/concentric-circles.html>). Briefly, an inner circle was arbitrarily selected, containing the most non-invasive intersections of the PDO. Concentric circles radiated every 12 µm from the inner circle encompassing the PDO. Strand number and distance were calculated per PDO. A minimum of 15 PDOs were quantified per condition.

*Surface area quantification*

HNSCC PDOs were treated with trypsin and 5,000 cells were seeded in 50 μL BME hanging drop. PDOs were cultured for 3 days and treated with or without 1 mg/mL DOX for a further 6 days in the presence of expansion media. Brightfield images were acquired on day 9 using the EVOS M500 microscope. To quantify PDO surface area, brightfield images were imported into FIJI. A threshold intensity was applied, and a binary mask was generated. The "Analyze Particles" function was used to measure the PDO surface area.

*BrdU incorporation*

PDOs were cultured in BME for 6 days and 10 μM BrdU (BD Biosciences) was added for 2 hours at 37°C. PDOs were fixed for 10 minutes with 4% PFA. Fixed samples were treated with 2 M HCl for 90 minutes at room temperature and washed 4 times with PBS before conjugated anti-BrdU and DAPI was added overnight at 4°C. PDO BrdU incorporation was assessed using the LSM800 x40 obj. The percentage of BrdU positive cells per PDO was quantified using FIJI (number BrdU positive nuclei per PDO/ total number of nuclei (DAPI) per PDO).

*Single-cell mRNA sequencing*

Single-cell mRNA sequencing was performed as previously described (6). PDOs were cultured in BME for 6 days and subsequently seeded into a Collagen matrix for a further 3 days. PDOs were treated for 3 minutes with Collagenase (Cat# C0130, Sigma-Aldrich) at 37 °C before 10 minutes trypsinization. Single cells were resuspended in FACS buffer (0.5 mM EDTA and 5% FCS) and individual viable cells (DAPI negative) were seeded into three 384-well plates per condition. Single-cell mRNA-sequencing was performed by Single Cell Discoveries (Utrecht, The Netherlands), and downstream analysis was processed, explored, and visualized using Cellenics® community instance (https://scp.biomage.net/) hosted by Biomage (https://biomage.net/). Briefly, cells with low-quality reads, as determined by cells exhibiting ≤1000 unique molecular identifiers (UMIs) and mitochondrial reads higher than 5%, were filtered out. MASS package (v. 7.3–56) was applied to determine the linear relationship between genes with at least one count, and the UMIs of each barcode. Next, the predicted gene number for each barcode was determined using a fitted model tolerance of 1–α, where α is 1 divided by the number of droplets in each sample and the predicted cells inside the upper and lower boundaries were retained. scDblFinder (R package v. 1.11.37) was used to determine the probability of droplets containing duplicate cells. A duplicate score of >0.5 was applied to remove these cells from further analysis. To integrate data, ‘Harmony’ was applied. Clustering was performed using Seurat’s implementation of the Louvain method. Adjusted p-values were determined using the Benjamini–Hochberg correction. A LogFC>0.4 and adjusted p-value <0.001 were selected.

*Gene-set enrichment analysis (GSEA) and Gene ontology*

Ranked GSEA was performed on all genes ordered by log2 fold-change using WebGestalt (7). Both Gene Ontology (GO) Molecular Function terms and Reactome pathway annotations were queried, and significance was assessed using Normalized Enrichment Scores (NES) and false discovery rate (FDR). In parallel, a subset of differentially expressed genes (|log2FC| ≥ 0.4, adj. p < 0.001) derived from the single-cell mRNA sequencing dataset was subjected to over-representation analysis (ORA) using Enrichr (8–10). For targeted analyses, the GO term “growth factor activity” (GO:0008083) was used to evaluate transcriptional expression of ligands in the single-cell mRNA sequencing dataset.

*TMA scoring*

TMA samples and patient databases were obtained with agreement from Ruud H. Brakenhoff, Amsterdam University Medical Center. All scoring was performed by two independent observers; If no consensus was reached, a third independent observer was used.

*Mouse studies*

2x10E6 T4, T5 or T8 cells were transplanted orthotopically into the buccal mucosa of recipient male RAG2-/-;IL2Rγc-/- immunodeficient mice (Envigo, RRID:IMSR_ENV:HSD-021). Mice were 15-20 weeks old at the start of the experiment and weighed 27–35 grams. Tumor growth was measured weekly using a digital, pressure sensitive caliper (Mitutoyo). Once palpable tumors developed (50 mm^3^), mice were randomized and half of the mice were switched from a standard chow to a DOX-containing diet (200 mg/kg; A155D70201; Ssniff, Bio services) to induce shRNA expression (*n* = 13 for both control and FER-iKD groups). For mice that received FER PROTACs, 1 mg/kg was intraperitoneally injected twice a week, whereas control mice received the solvent, DMSO 25%, Saline75%w/w (*n* = 10 for both DMSO control and FER PROTAC groups). Investigators were blinded to group assignments during tumor measurement and endpoint assessment. All mice were included in the study. Mice were sacrificed once the tumor volume exceeded 1000 mm^3^ or reached a general humane endpoint. All mice were included in the final analysis.

*Mouse protrusive index, proliferation, and metastasis quantification*

The protrusive index was quantified as previously described (11). Briefly, protrusive indices were calculated by dividing the perimeter by the convex hull of the tumor. Three independent regions of interest at the tumor invasive front from 10 individual primary tissue sections were quantified for each condition. Primary mouse tissue sections were quantitatively assessed for proliferation using Ki67 immunohistochemistry. QuPath version 0.4.4 was used to quantify 3 independent regions of interest (250 µm x 200 µm) at the tumor invasive front from 8 individual primary tissue sections for each condition. To assess tumor burden in the lungs of mice orthotopically transplanted with HNSCC PDOs, lung tissue sections were analyzed for GFP expression using immunohistochemistry stained for GFP. The total number of lung metastasis was quantified in 13 mice per condition.

*Statistical analysis*

Statistical analysis and graph generation were performed utilizing GraphPad Prism 8.0.1 (GraphPad Software; La Jolla, California, USA, RRID:SCR_002798). For comparisons between two independent groups, an unpaired two-tailed Student’s t-test was used. For non-normally distributed data, the Mann–Whitney U test or Kruskal–Wallis test was applied. T-tests were performed under the assumptions of normally distributed data, equal variances between groups and independent observations. Error bars represent the standard deviation (SD). The asterisks indicate significant differences. All experiments were repeated 3 times except for when stated in the figure legend.

For mouse experiments, tumor volume measurements were analyzed using a two-way mixed-effects model analysis of variance (ANOVA) in GraphPad Prism 8.0.1 (GraphPad Software; La Jolla, California, USA, RRID:SCR_002798) to assess the effects of treatment over time and to account for repeated measures within subjects. Survival data were analyzed using the Kaplan–Meier method, and statistical significance between survival curves was determined using the Mantel–Cox log-rank test.

All statistical analyses performed on the TMA cohort were conducted using IBM SPSS Statistics version 28.0 (SPSS Inc., Chicago, IL, USA, RRID:SCR_002865). Associations between categorical variables were examined using Pearson's correlation for clinical samples, and statistical significance was determined through a two-tailed test (sig. 2-tailed). Survival analysis was performed with the Kaplan–Meier method. Multivariable analysis was performed using Cox regression to determine the hazard ratios.

*Study Approval*

The collection of patient tissue for the generation of organoids has been performed according to the guidelines of the European Network of Research Ethics Committees (EUREC) following European, national, and local law. The Biobank Research Ethics Committee of the University Medical Center Utrecht (TCBio) approved the biobanking protocol: 12-093 HUB-Cancer according to the University Medical Center Utrecht (UMCU) Biobanking Regulation. All donors in this study signed informed-consent forms prior to participation and can withdraw their consent at any time, leading to the prompt disposal of their tissue and any derived material, as well as the cessation of data collection. All animal experiments were performed in accordance with local, National and European guidelines under permit AVD1150020209964 issued by The Netherlands Food and Consumer Product Safety Authority (NVWA) of the Ministry of Agriculture, Nature and Food.

**References**

1. E. Driehuis, *et al.*, Oral Mucosal Organoids as a Potential Platform for Personalized Cancer Therapy. *Cancer Discov* **9**, 852–871 (2019).

2. R. C. J. Schackmann, *et al.*, Cytosolic p120-catenin regulates growth of metastatic lobular carcinoma through Rock1-mediated anoikis resistance. *J. Clin. Investig.* **121**, 3176–3188 (2011).

3. S. Tavares, *et al.*, FER regulates endosomal recycling and is a predictor for adjuvant taxane benefit in breast cancer. *Cell Reports* **39**, 110584 (2022).

4. D. R. Stirling, *et al.*, CellProfiler 4: improvements in speed, utility and usability. *BMC Bioinform.* **22**, 433 (2021).

5. T. Koorman, *et al.*, Spatial collagen stiffening promotes collective breast cancer cell invasion by reinforcing extracellular matrix alignment. *Oncogene* **41**, 2458–2469 (2022).

6. P. D. Haughton, *et al.*, Differential transcriptional invasion signatures from patient derived organoid models define a functional prognostic tool for head and neck cancer. *Oncogene* **43**, 2463–2474 (2024).

7. J. M. Elizarraras, *et al.*, WebGestalt 2024: faster gene set analysis and new support for metabolomics and multi-omics. *Nucleic Acids Res.* **52**, W415–W421 (2024).

8. Z. Xie, *et al.*, Gene Set Knowledge Discovery with Enrichr. *Curr. Protoc.* **1**, e90 (2021).

9. E. Y. Chen, *et al.*, Enrichr: interactive and collaborative HTML5 gene list enrichment analysis tool. *BMC Bioinform.* **14**, 128 (2013).

10. M. V. Kuleshov, *et al.*, Enrichr: a comprehensive gene set enrichment analysis web server 2016 update. *Nucleic Acids Res.* **44**, W90–W97 (2016).

11. A. A. Khalil, *et al.*, A YAP-centered mechanotransduction loop drives collective breast cancer cell invasion. *Nat. Commun.* **15**, 4866 (2024).

**Supplemental Figures and Figure Legends**

**
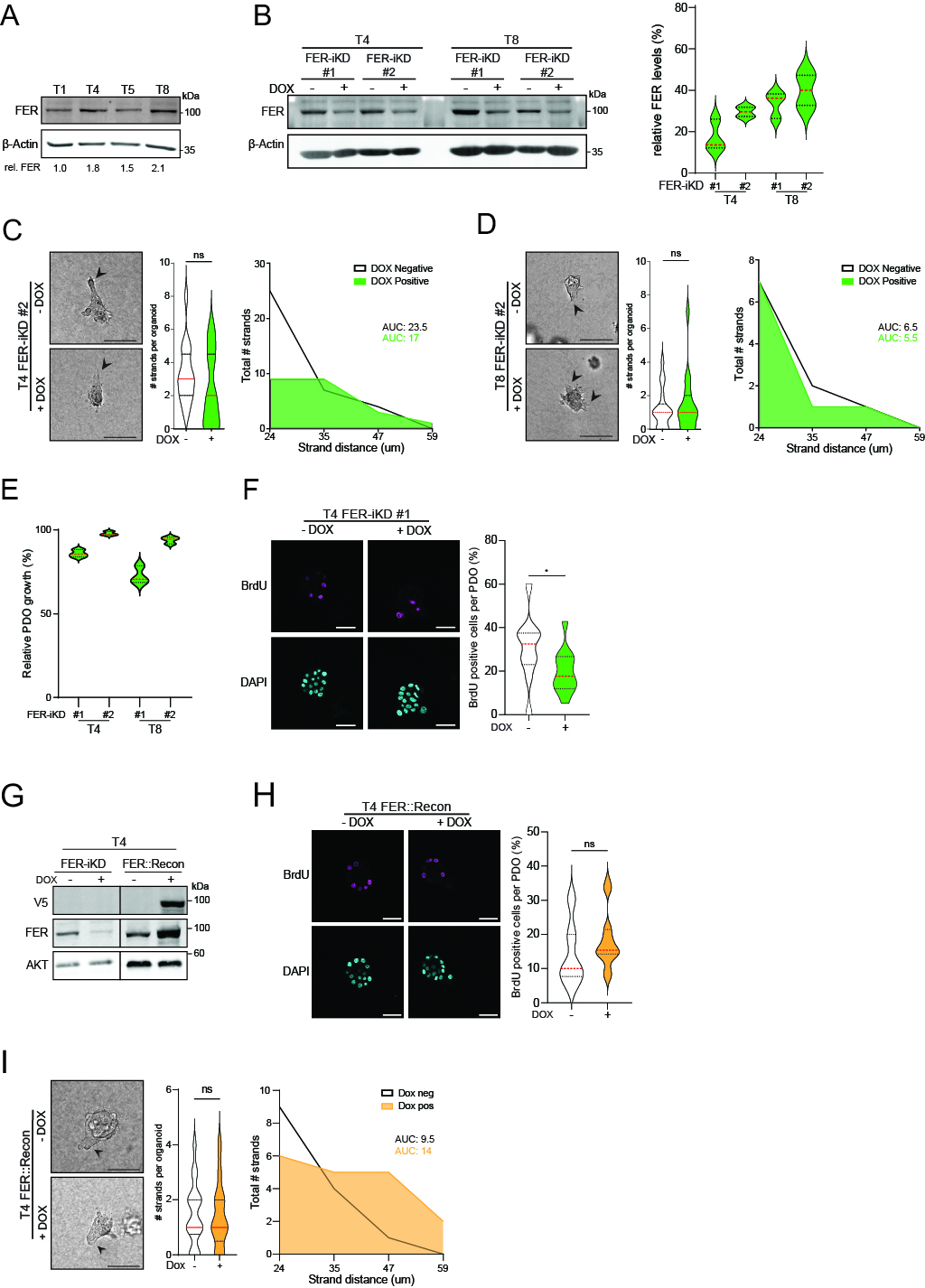
**

**Supplemental Figure 1** - FER controls invasive growth in HNSCC PDOs. **A**) Western blot analysis of T1, T4, T5 and T8 HNSCC PDOs, probed for FER and β-actin as loading control. Relative FER expression levels (FER/β-actin) is shown in red. **B**) T4 and T8 FER-iKD #1 and FER iKD #2 were cultured in the absence (-) or presence of DOX (+) and probed for FER and β-actin as loading control. FER-iKD efficiency (FER/β-actin) was quantified from 3 independent western blots for both hairpins and models (right violin plot). **C** and **D**) Brightfield images show the effect of FER-iKD #2 (+DOX) in T4 (C) and T8 (D). Scale bars: 50 µm. The number of invasive strands per organoid (violin plot) and the depth of the invasive strands (strand distance; line graph) were quantified from three independent experiments *(n* = 20 PDOs per condition). **E** and **F**) FER kinase impairs proliferation in HNSCC PDO. **E**) Growth assessment of T4 and T8, FER-iKD #1, and FER-iKD #2 PDOs were assessed by quantifying the surface area using ImageJ from three independent experiments. **F**) T4 FER-iKD #1 PDOs were assessed for proliferation using BrdU thymidine analogue incorporation. Immunofluorescence images show BrdU (purple) and DNA (turquoise). Scale bars: 50 µm. The violin plot displays the percentage of BrdU-positive cells per PDO from three independent experiments (*n* = 20 PDOs per condition). **G**-**I**) FER kinase reconstitution rescues proliferation and invasion. FER-depleted T4 PDOs were reconstituted for FER (FER::Recon) with DOX (G). V5-tag and FER kinase were probed on western blot samples to confirm rescue. AKT was used as a loading control. T4 FER::Recon PDOs were assessed for BrdU thymidine analogue incorporation and immunofluorescence labeled for BrdU and DNA (H). Scale bars: 50 µm. The violin plot displays the percentage of BrdU-positive cells per PDO from three independent experiments (*n* = 20 PDOs per condition). Brightfield images show the effect FER::Recon (+DOX) in T4. Scale bars: 50 µm (I). The number of invasive strands per organoid (violin plot) and the distance of invasive strands (line graph) were quantified from three independent experiments (*n* = 20 PDOs per condition). Error bars = SD; ns = non-significant; *p < 0.05. The Area under the curve (AUC) was employed to quantify differences in strand length between conditions.

**Supplemental Figure** **2** – Growth factor expression and FER mediated pathway control in HNSCC. **A)** T4 FER-iKD cells seeded in Collagen-I were subjected to single-cell mRNA sequencing. Heatmaps depict normalized Z-scores of growth factor-related ligands expressed in control (-DOX, *n* = 509) and FER knockdown (+ DOX, *n* = 424) T4 PDO. The x-axis represents log2 fold change and the y-axis represents –log10(adjusted p-value). **B)** Volcano plot of differentially expressed (DE) genes comparing T4 FER-iKD control and depleted cells. Vertical red lines mark the logFC cut-offs at ≥ 0.4 and ≤ −0.4. The horizontal red line marks the adjusted p-value cut-off (<0.001). Upregulated genes in FER-high cells are shown in blue, and downregulated genes in red. Selected genes of interest are labeled. **C and D)** Over-representation analysis (ORA) based on single-cell mRNA sequencing from the T4 FER-iKD PDO model seeded in Collagen-I identifies pathways associated with FER expression. The top 10 enriched pathways in Molecular Function (A) or Reactome (B) are shown in the bar graph. **E)** HNSCC primary tumor sections T1, T4, T5, and T8 (left panels) and the corresponding PDO models (right panels) were assessed for EGFR and MET expression. Scale bar: 100 µm.

**
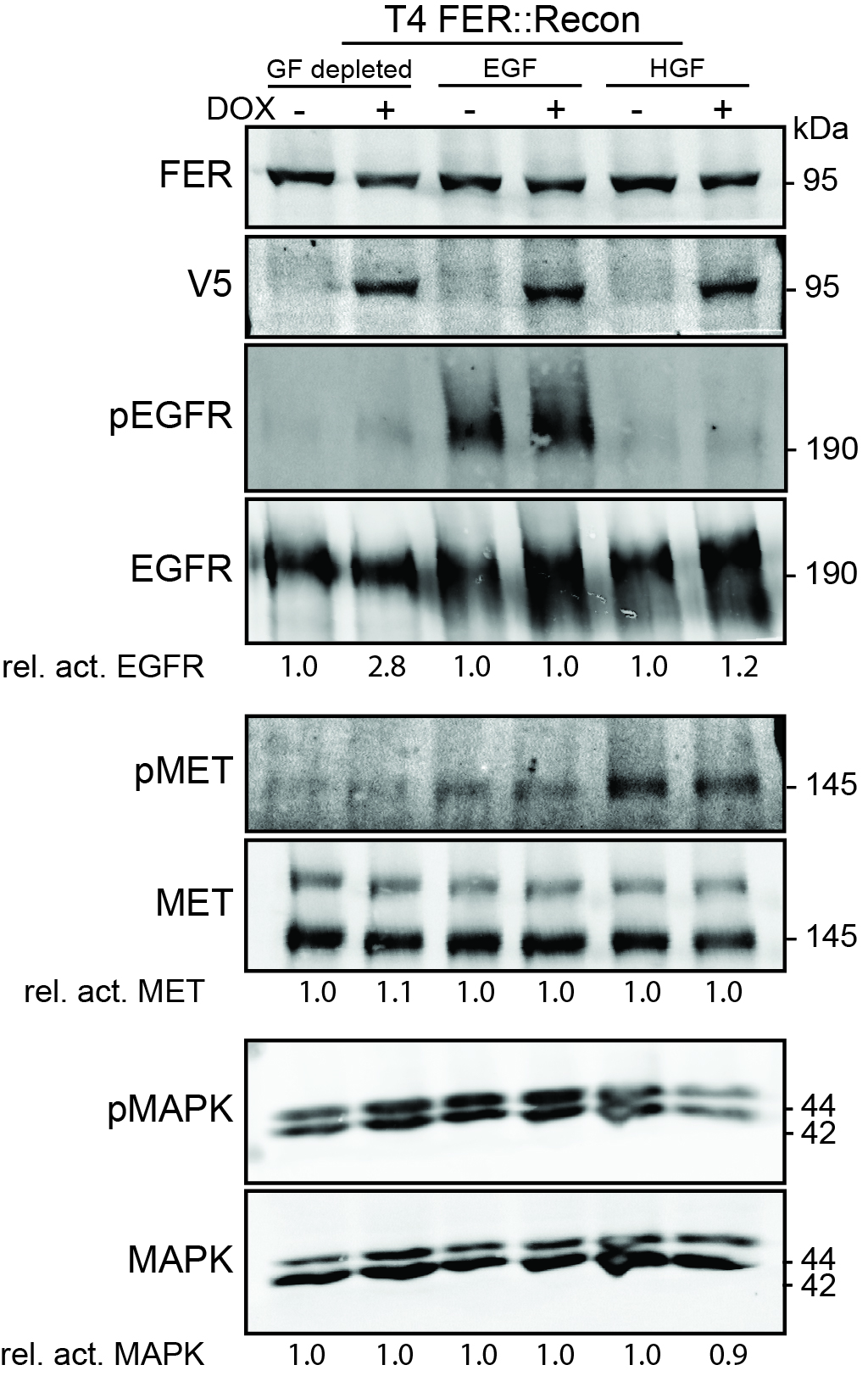
**

**Supplemental Figure 3** - FER reconstitution in FER knockdown HNSCC PDO T4 rescues EGFR and MET receptor signaling. Western blots of T4 FER::Recon system (DOX +) were either starved or stimulated with EGF or HGF and probed for FER, FER V5-tag, EGFR, pEGFR, MET, pMET, MAPK and pMAPK. Relative activation levels (phosphorylation/total) are shown.

**
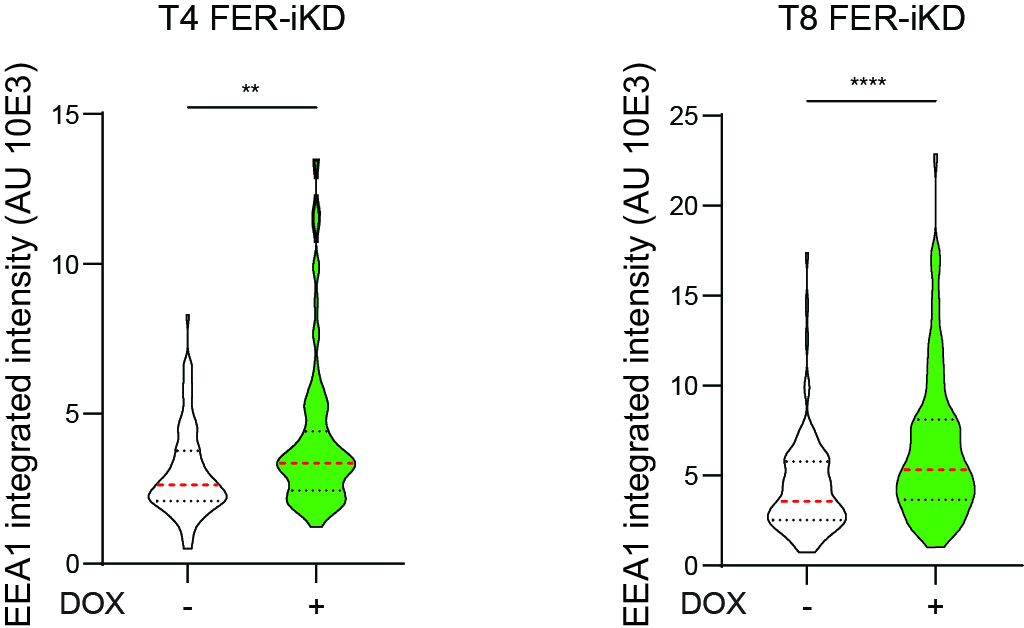
**

**Supplemental Figure 4** - FER depletion leads to an accumulation of early endosomes. FER-iKD PDO cells were cultured in growth factor-depleted media in the absence or presence of DOX and on Collagen-I-coated glass slides. The integrated intensity of EEA1 was assessed using immunofluorescence (Figure 4A and 4B left panels). Violin plots show the quantified EEA1 integrated intensity per cell from three independent experiments (*n* = 150 spots per condition). Error bars indicate SD; **p < 0.01, ****p < 0.001.

**
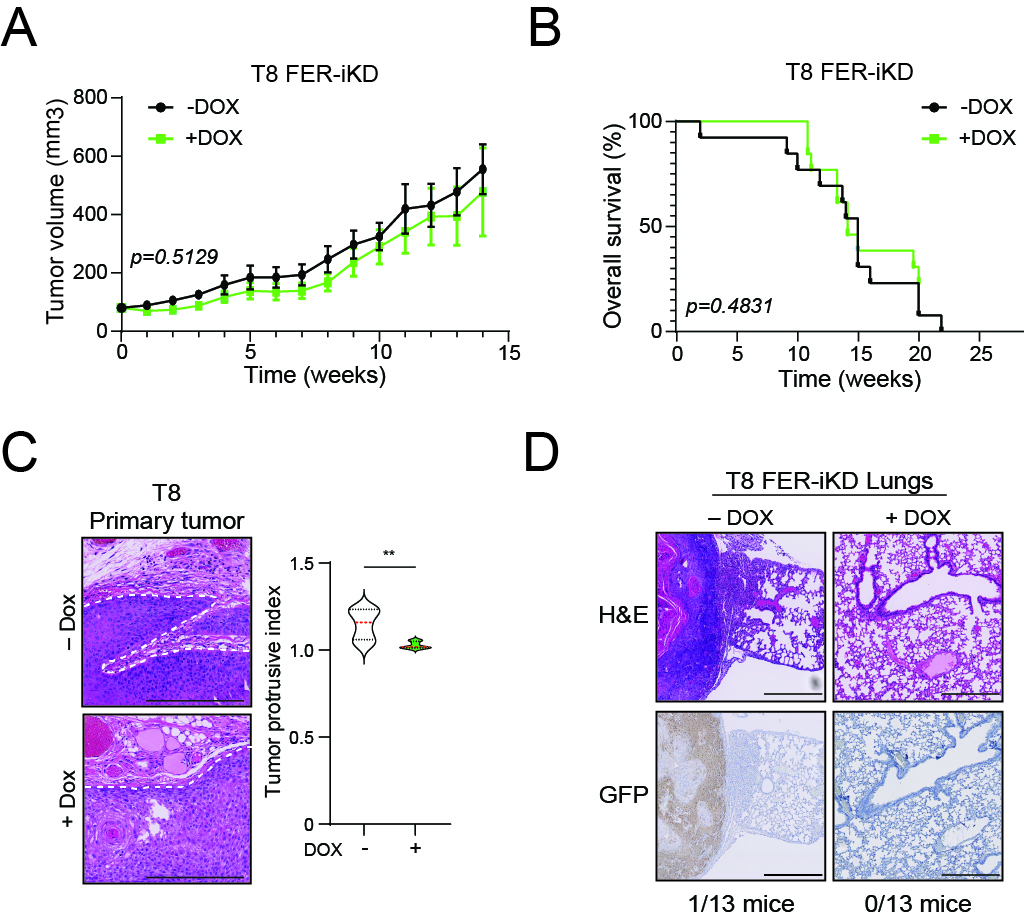
**

**Supplemental Figure 5 -** FER controls invasion and metastasis in T8 FER-iKD xenograft mouse models. **A**) T8 FER-iKD cells were transplanted into the buccal mucosa of recipient male mice. Upon the development of palpable tumors, mice were randomized and half of the mice were switched to a DOX-containing diet to induce FER knockdown. Values represent tumor volumes, error bars = s.e.m, n=13 for both the control (- DOX) and FER knockdown (+ DOX). **B**) Kaplan–Meier survival plot of the mice shown in (A). Animals were monitored and sacrificed once they reached the humane endpoint. **C**) H&E primary tissue sections depicting the effect of FER loss on invasive patterns into the surrounding stroma. White outlines indicate tumor boundaries. Scale bars: 200 µm. The violin plot shows the quantification of tumor protrusive indexes. **D**) Representative lung sections from control and FER knockdown mice were analyzed for the presence of tumor cells using immunohistochemistry for GFP. Scale Bars: 500 µm. Text below brightfield images indicate the number of mice with lung metastasis. Error bars indicate SD; **p < 0.01.


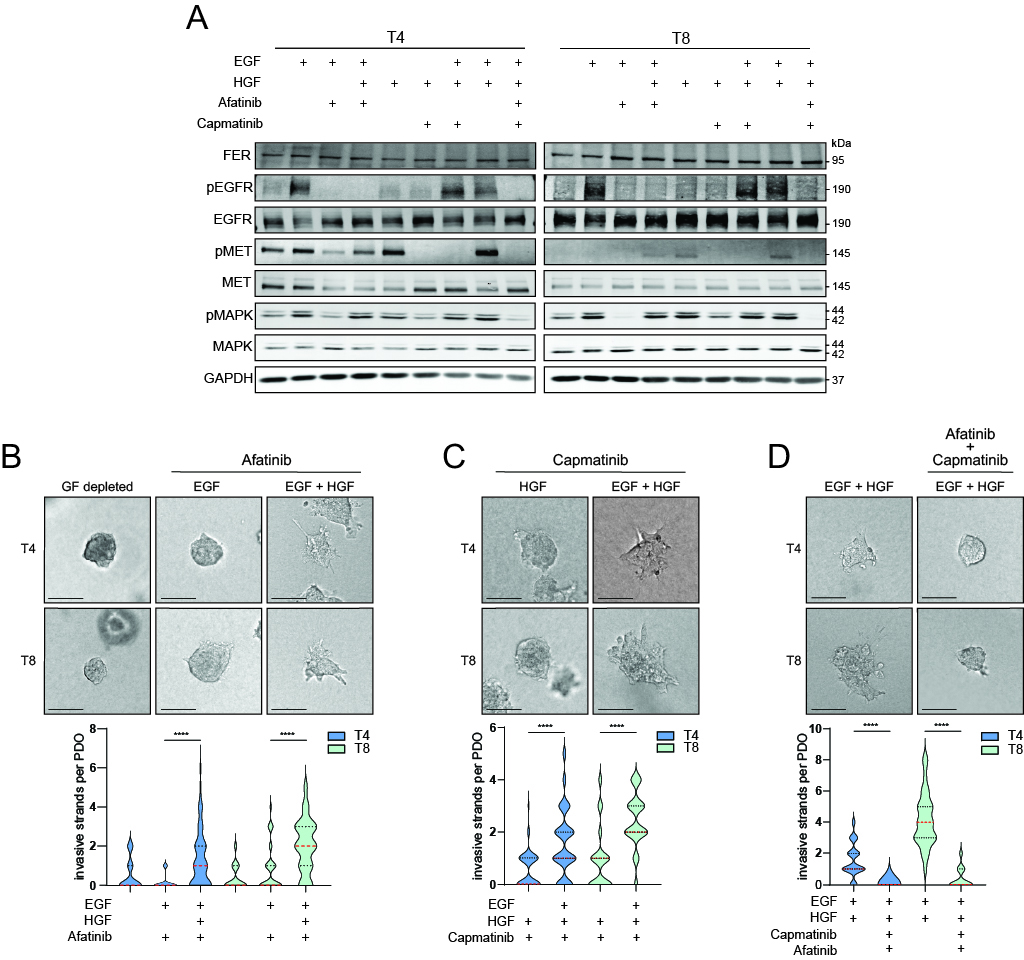


**Supplemental Figure 6** - HNSCC PDO models as a clinical proxy for studying GFR inhibition. **A**) Redundant receptor activation induces downstream activation. Shown are western blot analyses of T4 and T8 PDOs cultured in either growth factor depleted conditions or stimulated with EGF and/or HGF, in combination with or without Afatinib and/or Capmatinib. Blots were probed for EGFR, pEGFR (Tyr1068), MET, pMET (Tyr1234/5), MAPK, pMAPK (Thr202/Tyr204), and GAPDH as a loading control. **B-D**) Promiscuous GFR activation rescues inhibition of HNSCC invasion. Shown are brightfield images of T4 and T8 PDOs depleted of growth factors (left panels), treated with Afatinib in the presence of EGF (middle panels) and HGF (right panels)(B), or treated with Capmatinib in the presence of HGF (left panels) or EGF and HGF (right panels)(C), or stimulated with EGF and HGF (left panels) in the presence of Afatinib and Capmatinib (right panels)(D). Scale bars: 50 µm. Violin plots below the panels show the quantifications of the numbers of invasive strands per organoid from three independent experiments *(n* = 40 PDOs per condition). Error bars indicate SD; ****p < 0.001.


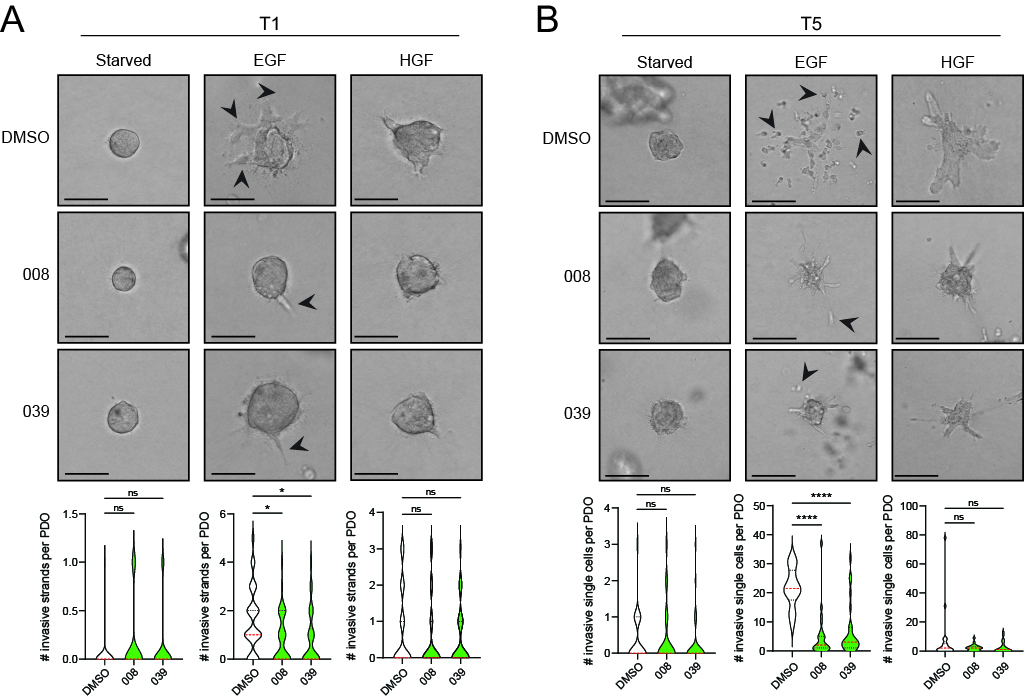


**Supplemental Figure 7** - FER-specific PROTAC compounds impair growth factor-mediated invasion in HNSCC PDOs.

**A** and **B**) FER-targeting PROTACs impair EGF and HGF-dependent invasion in HNSCC cells. T1 (A) or T5 (B) HNSCC PDO models were cultured in a Collagen-I matrix to allow invasive growth. HGF or EGF was added in the presence or absence of 50 nM 008 or 039 and assessed for invasion (DIC images). Arrowheads (black) indicate invasive strands. The number of invasive strands or invasive single cells per PDO (n = 40 per condition) was quantified for T1 and T5, respectively, across three independent experiments (bottom violin plots). Error bars indicate SD; ns indicates non-significant, *p < 0.05, ****p < 0.001.

**Supplemental movies**:

**Supplemental video 1 and 2**: FER kinase controls endosomal transport of EGF. T4 FER-iKD cells were cultured in the presence (Supplemental video 1) or absence (Supplemental video 2) of DOX on collagen-coated slides, and Alexa-555-conjugated EGF uptake was tracked for 10 minutes. Scale bars: 10 µm.
